# Octahedral gold-silver nanoframes with rich crystalline defects for efficient methanol oxidation manifesting a CO-promoting effect

**DOI:** 10.1038/s41467-019-11766-w

**Published:** 2019-08-22

**Authors:** Likun Xiong, Zhongti Sun, Xiang Zhang, Liang Zhao, Peng Huang, Xiwen Chen, Huidong Jin, Hao Sun, Yuebin Lian, Zhao Deng, Mark H. Rümmerli, Wanjian Yin, Duo Zhang, Shuao Wang, Yang Peng

**Affiliations:** 1 Soochow Institute for Energy and Materials Innovations, College of Energy, Soochow University, Suzhou, 215006 P. R. China; 2 Key Laboratory of Advanced Carbon Materials and Wearable Energy Technologies of Jiangsu Province, Suzhou, P. R. China; 3 State Key Laboratory of Radiation Medicine and Protection, School of Radiation Medicine and Protection, Soochow University, Suzhou, 215123 P. R. China; 4 Collaborative Innovation Center of Radiological Medicine of Jiangsu Higher Education Institutions, Suzhou, P. R. China

**Keywords:** Chemistry, Energy science and technology

## Abstract

Three-dimensional bimetallic nanoframes with high spatial diffusivity and surface heterogeneity possess remarkable catalytic activities owing to their highly exposed active surfaces and tunable electronic structure. Here we report a general one-pot strategy to prepare ultrathin octahedral Au_3_Ag nanoframes, with the formation mechanism explicitly elucidated through well-monitored temporal nanostructure evolution. Rich crystalline defects lead to lowered atomic coordination and varied electronic states of the metal atoms as evidenced by extensive structural characterizations. When used for electrocatalytic methanol oxidation, the Au_3_Ag nanoframes demonstrate superior performance with a high specific activity of 3.38 mA cm^−2^, 3.9 times that of the commercial Pt/C. More intriguingly, the kinetics of methanol oxidation on the Au_3_Ag nanoframes is counter-intuitively promoted by carbon monoxide. The enhancement is ascribed to the altered reaction pathway and enhanced OH^−^ co-adsorption on the defect-rich surfaces, which can be well understood from the d-band model and comprehensive density functional theory simulations.

## Abstract (web summary)

Direct methanol fuel cells are promising for clean, sustainable energy, but catalysts should be optimized. Here the authors construct ultrathin nanoframes with rich crystalline defects to increase electrocatalytic activity of gold for methanol oxidation, which is surprisingly promoted by carbon monoxide.

## Introduction

Direct methanol fuel cell (DMFC) is one of the promising clean and sustainable energy solutions for efficiently converting solar-converted and crop-based chemical energy into electric power^1,2^. As the basic anode reaction of DMFCs, electrochemical methanol oxidation (MOR) has thus received tremendous attentions^3–5^. As of today, platinum (Pt) based nanomaterials are still considered as the most efficient and widely implemented catalyst for MOR owing to their high affinity to methanol molecules and low activation barriers, both leading to enhanced kinetics of methanol oxidation. However, apart from its high cost, Pt also has very strong binding affinity to carbon monoxide (CO), one of the dissociation products from methanol oxidation, and even the exposure to a very small quantity of CO can severely poison the catalyst and thus drastically lower its MOR activity^6^. More recently, intense efforts have been devoted to advance the Pt-based MOR catalysts either by further elevating the specific catalytic activity, or by minimizing the Pt usage, through sophisticated structural and compositional manipulations^7–10^. However, the production cost and the inherent poisoning issue of Pt remain as challenges to be solved. Other researches have focused on Pd-based catalysts which also manifest fair MOR activities in alkaline electrolytes, but their oxidation to form PdO at similar potentials to MOR, together with the same issue of CO poisoning, limit practical implementation^11–14^.

While gold electrodes have long been studied for electrochemical oxidation of carbon monoxide^15–17^, more studies have been seen recently employing Au and Au-based nanomaterials as the catalysts for the oxidation of carbonaceous species such as alcohols and carboxylates^18–22^. MOR on these Au-based catalysts has been notoriously plagued with high electrochemical overpotentials and low redox kinetics. In fact, gold is the only metal that has an endothermic oxygen adsorption energy and hence is inert toward most oxidation reactions^23^. To overcome these issues, one important strategy coming to the forefront of Au catalyst innovations is the design and fabrication of multivariate nanocrystals with tailored geometries and tunable electronic structure, including mesoporous gold-silver networks, nanoporous gold, and hollow gold nanoparticles, etc^24–28^. In such context, Au nanoframes comprising only vertices and edges offer an appealing option to substitute Pt and Pd-based MOR catalysts by maximizing the utilization of active atoms, electrolyte-accessible surfaces, and surface electronic states^29,30^.

Among the limited reports in synthesizing Au-based nanoframes, a two-step template directed protocol has been typically employed involving the deposition of Au on the Ag templates followed by selective Ag etching by oxidants^31–33^. This additional etching step, however, greatly adds to the processing complexity and structural uncertainty, resulting in unsatisfactory material utilization and geometrical control. To circumvent this problem, herein we present a quick one-pot method for the preparation of ultrathin octahedral Au_3_Ag nanoframes with the edge diameters on the order of 5–10 nm and good geometrical uniformity. The reaction intermediates are closely monitored by scanning electron microscopy (SEM) and energy-dispersive X-ray spectroscopy (EDX) to unveil the underlying formation mechanism. Spherical-aberration-corrected TEM (Cs-TEM) is used to perceive lattice details on both the edges and vertices of the alloyed nanoframes, revealing rich crystalline defects as catalytically active sites. As a result, superior MOR activities to the Pt/C benchmark are demonstrated with a low-onset potential of 0.3 V (vs. RHE) and a high specific activity (SA) of 3.38 mA cm^−2^ (950 mA mg_(Au)_^−1^ in mass activity), which, to our best knowledge, are among the highest seen for all Au-based electrocatalysts reported so far (Supplementary Table 8). Moreover, our results show that on the surface of Au_3_Ag nanoframes CO plays a counter-intuitive role in promoting, rather than poisoning, the methanol oxidation reaction. Such promoting phenomenon is in accordance with Rodriguez’s implication about alcohol electro-oxidation on the Au surface in aqueous phases^34^.

## Results

### Temporal study on the formation of Au–Ag nanoframes

**Fig. 1:**
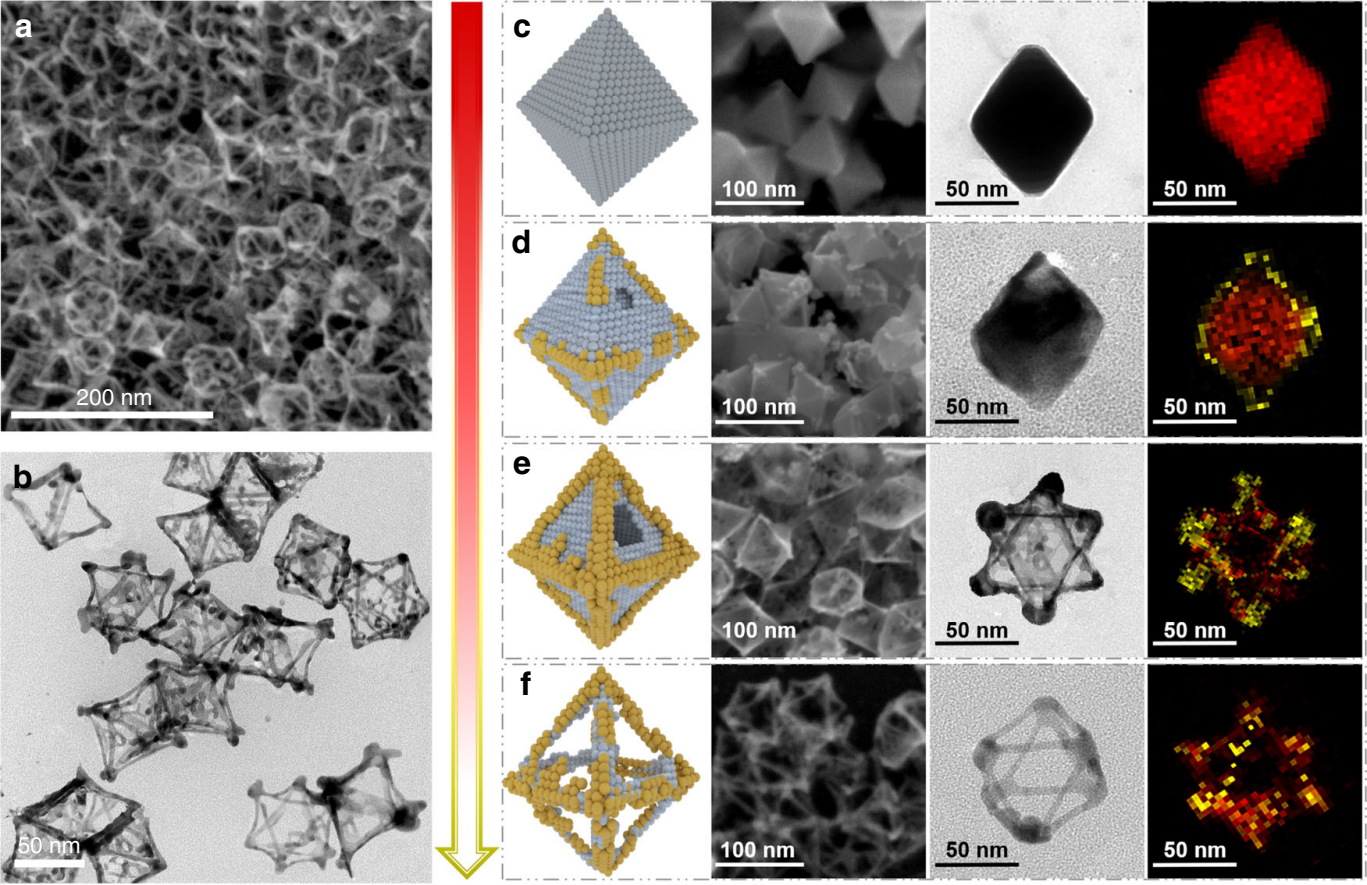
Morphological evolution of Ag nanoparticles to Au_3_Ag nanoframes. **a** Scanning electron microscopy (SEM) and **b** transmission electron microscopy (TEM) overview of Au_3_Ag NFs showing high structural uniformity. The schematic diagram, SEM, TEM, and energy-dispersive X-ray (EDX) mapping images of **c** Ag NPs collected at the 36th min before HAuCl_4_ addition, **d** AuAg_12_ NPs collected at the 38th min after HAuCl_4_ addition, **e** Au_2_Ag NCs collected at the 42th min, and **f** Au_3_Ag NFs collected at the 46th min

Ultrathin octahedral Au–Ag nanoframes with high geometrical uniformity (Fig. 1a, b) were synthesized under nonaqueous condition using chloroauric acid (HAuCl_4_) and silver nitrate (AgNO_3_) as the metal precursors, cetyltrimethylammonium bromide (CTAB) as the structure-directing and complexing agent, octadecylamine (ODA) as the solvent and surfactant, and cuprous chloride (CuCl) as the co-reducing agent (see the Supplementary information for experimental details). To understand the formation mechanism, the growth of the alloyed Au–Ag nanoframes was monitored by both SEM and TEM along the reaction course (Fig. 1c–f). Note that HAuCl_4_ was added at the 36th min from the beginning of the reaction, and prior to that octahedral Ag nanoparticles had already been formed through the reduction of AgNO_3_ by ODA and CuCl in the presence of CTAB. Four samples were then collected at the 36th (right before the injection of HAuCl_4_), 38th, 42nd, and 46th min of the reaction for structural and compositional analysis.

Figure 1c present the schematic diagram, SEM, TEM, and EDX mapping images of the octahedral Ag nanoparticles obtained from the first stage of the reaction, in which CuCl serves as the co-reducing and structure-directing agent for the formation of polyhedrons^35,36^. More importantly, the bromide ions from CTAB can lower the reduction rate of Ag^+^, promoting the preferential growth of the (111) facets that possess the lowest surface energy (γ_111_ < γ_100_ < γ_110_), and thus lead to the exclusive formation of octahedrons^37,38^. The resulted octahedral Ag nanoparticles (Ag NPs) have clean and smooth surfaces with an average edge length of 60.8 ± 4.1 nm (Supplementary Fig. 1a). Further crystallographic characterizations by X-ray diffraction (XRD) and Cs-TEM indicate the Ag nanoparticles are single crystalline with minimal defects (Supplementary Figs. 2 and 3).

Upon the instant injection of HAuCl_4_, Au^3+^ was first reduced to Au^+^ (rather than Au^0^), which can be stabilized by the bromide ions from CTAB to form Au–Br complexes^39,40^. Comparing to Au^3+^, the oxidizing potency of Au^+^ is drastically weakened, leading to a more controlled galvanic reaction with Ag^0^. During the second reaction stage as shown in Fig. 1d, the reactive Au^+^ were first deposited onto the edges and vertices of Ag octahedrons via galvanic replacement. This selective deposition is synergistically enabled by two obvious facts. First, the low-coordination states of the Ag atoms located at the edges and vertices make them more reactive than those on the flat terraces. Second, the Ag (111) facets are capped by ODA and CTAB, resulting in significantly passivated surfaces. The products obtained from this stage are bimetallic octahedral nanoparticles, with the average edge length increased to 65.9 ± 3.9 nm at the 38th minute (Supplementary Fig. 1b).

The deposited Au accumulating on the edges and vertices of the Ag octahedra gradually forms a natural defense, effectively shielding the underlying Ag atoms from being further oxidized as the reaction goes on. Thus, the newly reduced Au^+^ tend to attack those less-passivated areas, leading to the formation of pinholes on the crystal surfaces. A galvanic cell is thus formed between the outer Au cathode and the inner Ag anode, whereat silver atoms are oxidized and each provides one electron to reduce one Au^+^^41^. Since the outside Ag (111) facets are capped by ODA and CTAB, the released electrons tend to migrate to the edges and vertices, where AuCl^2−^ are reduced to Au (the cathode reaction). The liberated Ag^+^ ions, after reacting with Cl^−^, and Br^−^, are retained in the soluble form by complexing with ODA and CTAB. In addition, the dissolution of Ag^+^ ions promotes the local concentration of Cl^−^ and Br^−^, further in favor of the pinhole growth via an autocatalytic process^39^. As the cavities inside the silver octahedra continuously grow, hollow Au–Ag cages with surface pinholes were formed as evidenced by both TEM and SEM images taken at the 42nd minute (Fig. 1e). Meanwhile, the edges of the Au–Ag nanocages increased to 71.3 ± 4.4 nm (Supplementary Fig. 1c).

Concurrent with the galvanic replacement reactions, the deposited Au and the underlying Ag atoms on the edges and vertices alloy with each other, driven by the high interdiffusion rate of Au and Ag at 125 °C and the formation of energetically stabler alloyed phases. As the reaction continues, the inner Ag atoms are gradually etched away until the final collapse of the crystal surface, which is passivated from outside by ODA and halide ions. Instead, the edges and vertices of the nanocrystals are capable of surviving through the galvanic reactions, thanks to the stabilized Au–Ag alloy layer. As a result, well-defined Au–Ag nanoframes with ultrathin edges were obtained at the 46th min when the reaction was promptly stopped (Fig. 1f). The average size of the Au–Ag nanoframes obtained from this stage is 71.8 ± 4.1 nm (Supplementary Fig. 1d), similar to that of the nanocages from the third stage. We note that further extending the reaction time might lead to undesired structural damage through dealloying (Supplementary Fig. 4).

As an outcome of the above temporal study, bimetallic Au–Ag nanostructures with morphologies of nanoparticles, nanocages and nanoframes can be obtained from the same one-pot reaction by terminating it at different stage. The atomic ratios of Au and Ag in each of these nanostructures were quantified by EDX, X-ray photoelectron spectroscopy (XPS), and inductively coupled plasma optical emission spectroscopy (ICP-OES), showing similar values to each other (Supplementary Table 1, Supplementary Fig. 5). Specifically, the Au–Ag nanoparticles have an Au/Ag ratio of approximately 1:12, whereas that of the nanocages is about 2:1 due to the removal of Ag atoms inside the nanoparticles. As for the Au–Ag nanoframes, the atomic ratio of Au/Ag is further increased to about 3:1 as a result of the loss of surface atoms. Consequently, based on the composition analysis, the Au–Ag nanostructures obtained from different reaction stages are respectively designated as Ag NPs, AuAg_12_ NPs, Au_2_Ag NCs and Au_3_Ag NFs, which will be used throughout the rest of discussions.

### Lattice details of Au3Ag nanoframes

**Fig. 2:**
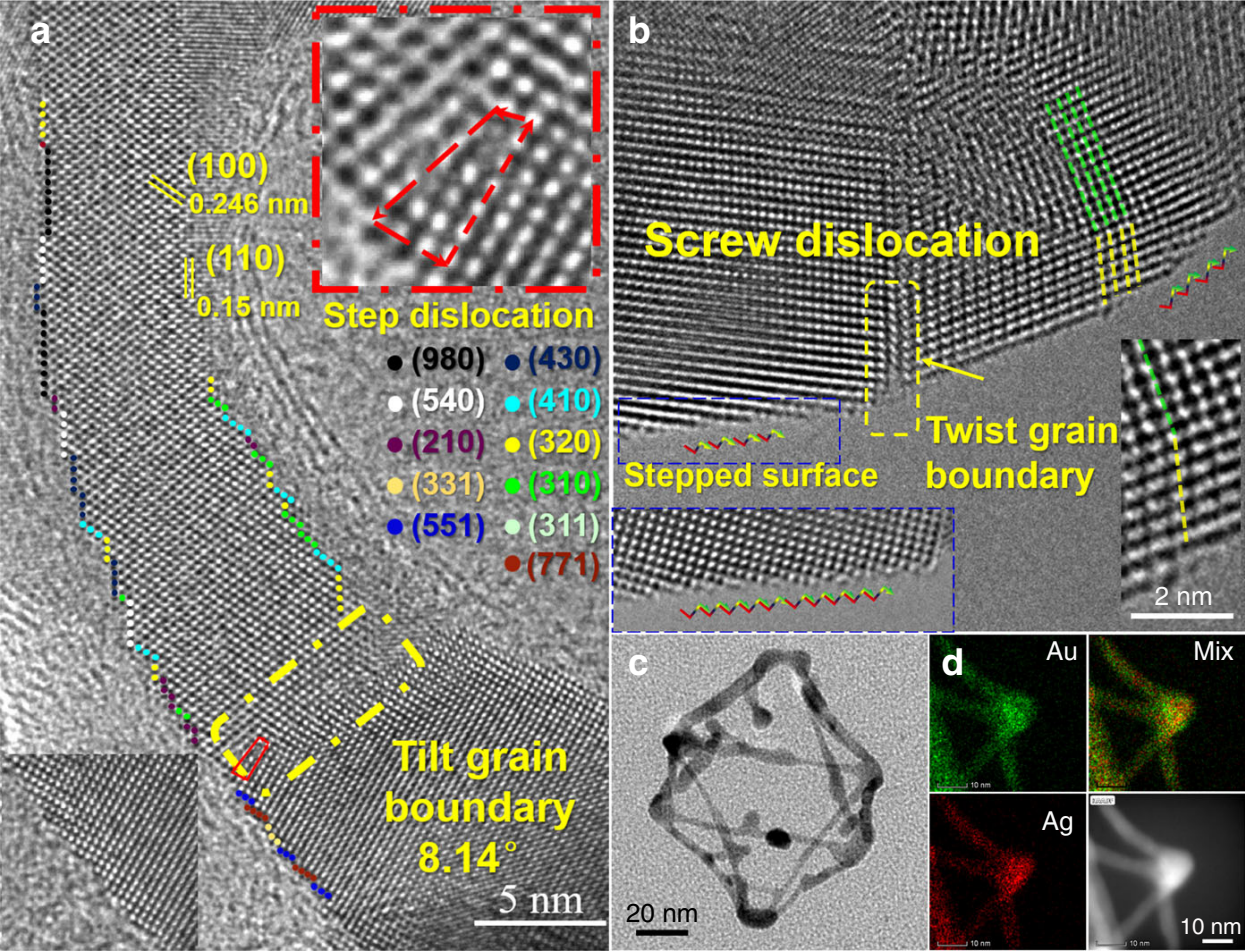
Atomic structure of Au_3_Ag nanoframes revealing rich crystalline defects. **a** High-resolution lattice details on one of the edges displaying an edge dislocation, high-index surface atoms, point defects, and tilt grain boundaries. **b** High-resolution lattice details on one of the vertices displaying stepped surface atoms, screw dislocations, and twist grain boundaries. **c** Transmission electron microscopy (TEM) overview of the nanoframe. **d** Energy-dispersive X-ray (EDX) mapping images of Au and Ag on a vertex connecting four edges

As the physiochemical properties of the alloyed nanoframes are highly correlated to their atomic arrangement and surface orientation, Cs-TEM was employed to characterize the detailed lattice configuration of Au_3_Ag NFs in high spatial resolution. Various sections chosen at the edges and vertices were elaborately inspected (Fig. 2, Supplementary Fig. 6). Figure 2a shows a thin edge of a nanoframe viewed from the (111) direction, expressing lattice fringes comprising the (100) and (110) planes. On its curved contours, the surfaces are full of zigzag atomic steps with the high-index facets denoted by different colors using the Microfacet notation developed by Somorjal et al.^42,43^. Beneath the surface, defects of edge dislocation, caused by the insertion of additional planes into the otherwise self-contained lattice, can be found as the one highlighted by the red dotted box shown in the upper inset of Fig. 2a. Further inside the edge dislocation, point defects of missing atoms are clearly discernable. The formation of edge dislocations further gives rise to lattice distortion, resulting in the development of low-angle tilt grain boundaries (yellow dotted box in Fig. 2a).

On the vertex of the nanoframe shown in Fig. 2b, a typical screw dislocation is present, probably resulted from local asymmetrical heating during Au deposition^44^. Upon intersecting with the crystal surface, the screw dislocation creates multiple step edges, propagating into self-perpetuating spirals. Stacking fault is another commonly observed defect with some in parallel with the Au (111) surface shown in Supplementary Fig. 6c. Besides, numerous grain boundaries are scattered throughout the vertices of the nanoframe. In general, the high-resolution analysis made here by CS-TEM unveiled abundant structural defects on the Au_3_Ag NFs, including atomic steps, dislocations, vacancies, and grain boundaries, which should effectively alter the surface electronic states, and thereby bring in dramatically different properties and activities from their bulk counterparts.

The high-resolution EDX mapping images acquired under the HAADF-STEM mode indicate on the Au–Ag NFs Au atoms are preferentially located atop the Ag core (Fig. 2d). By comparing the Au and Ag elemental mapping images (where a vertex connecting four edges is displayed), we note not only the intensity of the Au signal is higher, but also is the area it covers. This becomes even more obvious by overlaying the Au and Ag images together, affirming the surfaces are majorly constituted by Au atoms. It is well-known in order to minimize the overall Gibbs free energy, the inter-diffusion and segregation of alloyed elements can occur in most multi-component materials under suitable environmental bias, such as the high temperature synthetic conditions employed here. Therefore, the atomic inter-diffusion and thermal motion serve as the driving force for creating vacancies, steps and dislocations on the Au_3_Ag NFs^45,46^.

### Spectroscopic analysis on structural and surface states

**Fig. 3:**
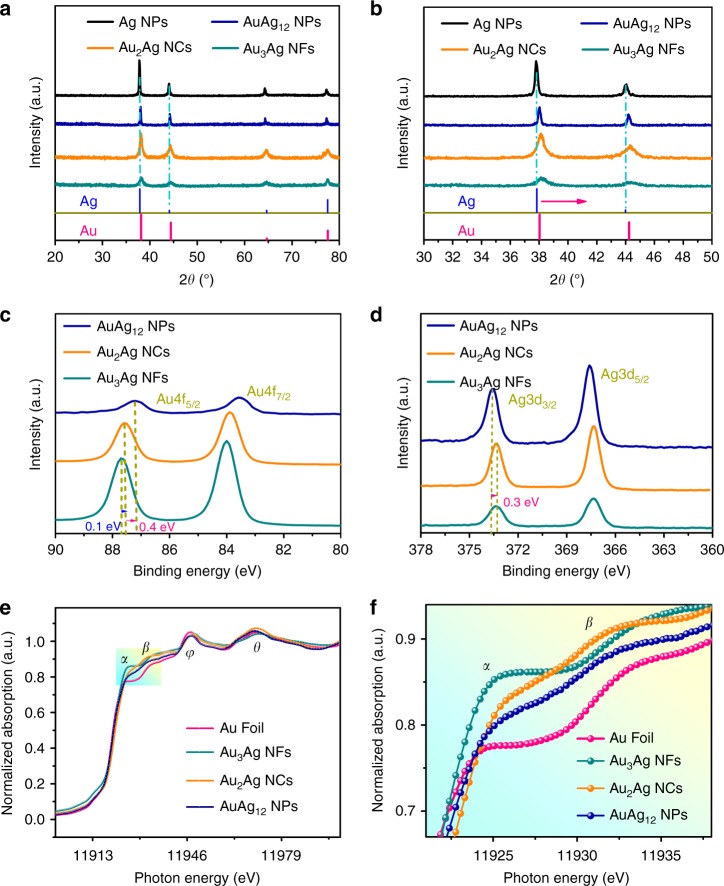
Spectroscopy analysis on the structural evolution for all AuAg nanostructures. **a** X-ray diffraction (XRD) and **b** zoom-in spectra of Ag NPs, AuAg_12_ NPs, Au_2_Ag NCs, and Au_3_Ag NFs. **c** X-ray photoelectron spectroscopy (XPS) Au 4f and **d** Ag 3d spectra of AuAg_12_ NPs, Au_2_Ag NCs, and Au_3_Ag NFs. **e** X-ray absorption near-edge structure (XANES), and **f** zoom-in spectra obtained from the Au L_3_-edge absorption of AuAg_12_ NPs, Au_2_Ag NCs, and Au_3_Ag NFs in comparison to the gold foil

XRD analysis was carried out to characterize the evolution of crystallographic structure for the above Au–Ag nanostructures collected from different reaction stages (Fig. 3a, b). Since Au and Ag have very similar face-centered cubic (fcc) crystal structure, their four main diffraction peaks indexed to the (111), (200), (220), and (311) planes are very close to each other. However, a careful examination will find out there still exist subtle differences between Au and Ag in the peak positions of (111) and (200) planes (2*θ*_Au (111)_ = 38.10° vs. 2*θ*_Ag (111)_ = 37.93°, 2*θ*_Au (200)_ = 44.37° vs. 2*θ*_Au (200)_ = 44.142°). As shown in Fig. 3a, XRD patterns of the four samples collected at the 36th, 38th, 42nd, and 46th min of the reaction, namely Ag NPs, AuAg_12_ NPs, Au_2_Ag NCs, and Au_3_Ag NFs, present decreased crystallinity with increasing reaction time, which is in accordance with the alloying process. A closer inspection on the (111) and (200) peaks clearly reveals their shift to higher 2*θ* angles as the Au content increases in the nanostructures (Fig. 3b), corroborating the above elemental analysis by EDX, XPS, and ICP-OES.

To probe the surface electronic states and local coordination of Au and Ag atoms, XPS and X-ray absorption fine structure (XAFS) analyses were performed on the samples of AuAg_12_ NPs, Au_2_Ag NCs, and Au_3_Ag NFs. As shown in Fig. 3c, the XPS Au 4f peaks shift to higher binding energies as the Au–Ag nanostructures transform from nanoparticles to nanocages and further to nanoframes, with a total displacement of 0.5 eV. This upshift of binding energies indicates that in comparison to Au_2_Ag NCs and AuAg_12_ NPs, the Au d orbitals of Au_3_Ag NFs are more depleted since the binding-energy shift strongly correlates to the Coulomb integrals of *F*_4f,5d_^47,48^. On the other side, the Ag 3d peaks shift slightly to lower binding energies with a total displacement of 0.3 eV from AuAg_12_ NPs to Au_3_Ag NFs (Fig. 3d). These observations suggest that Au in the Au_3_Ag NFs are positively polarized (Au^δ+^) and their d-band center shifts up toward the Fermi level (*E*_F_), whereas the Ag d bands shift down relative to *E*_F_^49–51^. Interestingly, this observation is opposite to the charge transfer between Au and Ag induced by the ligand effect in respond to the higher work function of Au, inferring that compared to the structural attributes the ligand effect is negligible here. To further resolve this inconsistency, all samples were then subjected to analyses of the Au L_3_-edge X-ray absorption near-edge structure (XANES).

Figure 3e shows that all Au–Ag nanostructures display similar resonance patterns in the normalized XANES spectra as that of the Au foil with four characteristic peaks, indicating they all have Au-like coordination environments. Importantly, the leftmost adsorption peak (peak *α*) refers to the intensity of resonance at the threshold (white-line peak) associated with the dipole transition from 2p_3/2_ to 5d_5/2,3/2_, reflecting the unoccupied density of d states at the Fermi level. As magnified in Fig. 3f, the white-line peak intensity follows the order of Au foil < AuAg_12_ NPs < Au_2_Ag NCs < Au_3_Ag NFs, suggesting a corresponding increase in d orbital depletion, which is in good agreement with the above XPS results. Previous studies have shown the ligand effect has negligible impact on the surface reactivity beyond a few atomic layers^52,53^, it is therefore, surmised the surface structure, lattice distortion and defects play a dominating role in increasing the observed d-hole population of Au. Furthermore, both the FT-EXAFS of the Au foil and Au_3_Ag NFs display doublets in the region of 1.8–3.4 Å, comprising a low-R peak (2.0–2.2 Å) and a high-R peak (2.4–2.9 Å) that can be ascribed to the nearest Au–Au bonding (Supplementary Fig. 7)^54,55^. This enables us to further deduce quantitative structural parameters shown in Supplementary Table 2 regarding the local Au bonding environment in Au foil and Au_3_Ag NFs. Compared to the bulk Au foil, Au_3_Ag NFs exhibit significant reduction in the coordination number of nearest Au neighbors from 12 to 9.4, confirming most Au atoms in Au_3_Ag NFs are in surface or defect states. Further analysis on the resulted bond lengths suggests a lattice contraction in the Au_3_Ag NFs (2.86 Å for Au foil vs. 2.84 Å for Au_3_Ag NFs), owing again to the existence of abundant defects in the alloyed structure. This argument is further evidenced by the relatively large Debye–Waller factor (*σ*^2^) obtained for Au_3_Ag NFs, corroborating the increased disorder in the Au–Au shell as a result of the defective lattice structure, echoing previous observations made by Cs-TEM.

Complementary to the above spectroscopy analyses, cyclic voltammetry (CV) was taken to further correlate the microstructure with surface redox potentials for Au_3_Ag NFs, Au_2_Ag NCs, and AuAg_12_ NPs. All CVs acquired in 0.1 M HClO_4_ exhibit two major anodic peaks, attributed to the oxidation of surface Ag and Au atoms, respectively (Supplementary Fig. 8). The sample of Au_3_Ag NFs exhibits the largest Au oxidation peak located at 1.43 V (vs. RHE), lower than those of Au_2_Ag NCs (1.59 V) and AuAg_12_ NPs (1.80 V). It also shows a smaller Ag oxidation peak at the lowest potential (0.56 V) with respect to Au_2_Ag NCs (0.58 V) and AuAg_12_ NPs (0.68 V), corroborating that Au_3_Ag NFs possess more unsaturated and active surface atoms that are easier to oxidize, in good agreement with the EXAFS results.

### Structure-dependent MOR performance

**Fig. 4:**
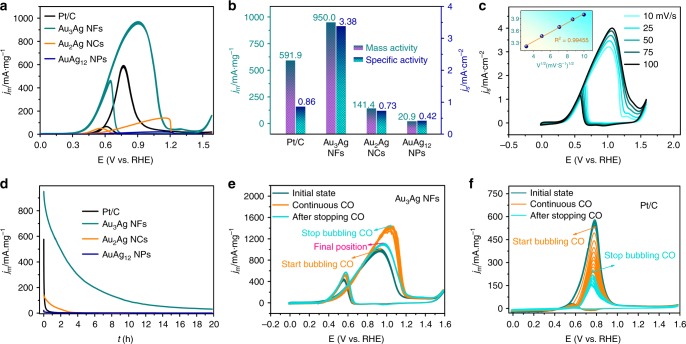
Electrocatalytic performances of Au–Ag nanostructures. **a** Cyclic Voltammetry (CV) curves taken at a sweep rate of 20 mV s^−1^ for all examined catalysts. **b** The corresponding histogram of mass and specific activities. **c** CVs of Au_3_Ag NFs taken at various scan rates. The inset shows a linear response of current density with respect to the square root of scan rates. **d** Chronoamperometric *i*–*t* curves of all examined catalysts up to 20 h. **e** Evolution of CV curves for Au_3_Ag NFs with the CO supply turned on and off for a continuous 10 cycles. **f** Evolution of CV curves for Pt/C with the CO supply turned on and off for a continuous 10 cycles. The methanol oxidation reactions were performed in deoxygenated solutions of 2 M CH_3_OH and 0.5 M KOH

The electrocatalytic activities of Au_3_Ag NFs, Au_2_Ag NCs, and AuAg_12_ NPs toward MOR were evaluated against the benchmark of commercial 20% Pt/C. Figure 4a shows the CVs of all tested samples in N_2_-purged 0.5 M KOH solution with 2 M methanol, exhibiting characteristic MOR behaviors with both forward and backward oxidation peaks. For Au_3_Ag NFs, the forward scan reveals a MOR onset potential at 0.30 V and peak oxidation current at 0.92 V. In the backward sweep, a second oxidation peak is observed at 0.65 V, owing to the removal of residual carbonaceous species formed in the forward scan^56^. For comparison, the commercial Pt/C exhibits a higher onset potential at 0.4 V but lower potential of peak current at 0.78 V. Nevertheless, the peak current density of Pt/C (591.9 mA mg_pt_^−1^@0.78 V) is still lower than the current density of Au_3_Ag NFs at the same potential (814.5 mA mg_Au_^−1^@0.78 V), not to mention its peak current density of 950 mA mg_Au_^−1^@0.92 V. By contrast, Au_2_Ag NCs and AuAg_12_ NPs present much higher peak–current potentials at 1.15 and 1.21 V, respectively, with the corresponding peak current densities of 141.4 and 20.9 mA mg_Au_^−1^. Consequently, the mass activity (MA) of Au_3_Ag NFs is 1.6, 6.7, and 45.5 times that of commercial Pt/C, Au_2_Ag NCs, and AuAg_12_ NPs, respectively. After normalized to the specific surface area (quantified by CO stripping in Supplementary Fig. 9), the SA of Au_3_Ag NFs is 3.38 mA cm^−2^, which is 3.9, 4.6, and 8.0 times that of the commercial Pt/C, Au_2_Ag NCs, and AuAg_12_ NPs, respectively (Fig. 4b). In addition, the catalytic MOR performance of the octahedral Ag NPs obtained at the 36th min before the addition of HAuCl_4_ was also examined, and the results show no obvious difference between the CV curves obtained with and without methanol (Supplementary Fig. 10), suggesting that the Au phase in the bimetallic nanostructures plays a dominant role in catalyzing the methanol oxidation.

To confirm the observed high catalytic activities are indeed due to the oxidation of methanol, rather than any residual organics, we performed concentration-dependent studies on the Au_3_Ag NFs catalyst by varying the concentration of methanol in 0.5 M KOH (Supplementary Fig. 11). The measured current increases with the methanol addition until a concentration of 2 M, beyond which the MOR kinetics start to decrease. This observation strongly suggests a trade-off between the reactant concentration and the permittivity of the electrolyte exerting on the reaction rate. Therefore, the optimal methanol concentration of 2 M was used throughout this work. What’s more, CVs acquired at different scan rates on Au_3_Ag NFs show that the anodic peak current is linearly proportional to the square root of scan rate, indicating the electrocatalytic oxidation of methanol is governed by a diffusion-controlled process (Fig. 4c), which is greatly facilitated by the highly exposed active sites of Au_3_Ag NFs. To further identify the activity origin, we deliberately destroy the framework of the nanoframes through ball milling, and found out the MOR MA declined only slightly from 950 to 914 mA mg_(Au)_^−1^ at similar voltages (Supplementary Fig. 12). CS-TEM images indicate after ball milling the crystalline defects were mostly unaffected (Supplementary Fig. 13), but the frameworks were turned into thin wires (Supplementary Fig. 14). This offers extra evidence to support the enhanced MOR activity is indeed majorly originated from the abundant crystalline defects.

To assess the catalytic stability, chronoamperometric *i*–*t* curves were taken for all Au–Ag nanostructures, in comparison with the commercial Pt/C, at their peak–current potentials (Fig. 4d). The Au_3_Ag NFs exhibited a gradual decrease in mass activity, retaining 502.1 mA mg^−1^ after 2 h and 32.2 mA mg^−1^ after 20 h. By contrast, the Au_2_Ag NCs and AuAg_12_ NPs showed a much faster activity decay, with only 34.2 and 3.2 mA mg^−1^ retained after 2 h, respectively. As for the Pt/C, the mass activity was quickly lost and almost diminished to 0 mA mg^−1^ after just 1 h, likely due to the heavy accumulation of carbonaceous intermediates poisoning the active sites on catalyst surface. This point of view can be further understood from the comparison of I_f_/I_b_, the ratio of the forward anodic peak current (*I*_f_) to the backward anodic peak current (*I*_b_), which is used to assess the tolerance of the catalyst to the poisoning carbonaceous intermediates such as carbon monoxide and formate. A lower *I*_f_/*I*_b_ value signifies the capability of the catalyst to eliminate residual carbonaceous species from surface, whereas a high *I*_f_/*I*_b_ value suggests its incompetence on this matter^27^. Specifically, the Au_3_Ag NFs has the lowest *I*_f_/*I*_b_ of 2.5, in comparison to 9.2, 2.9, and 5.6 for the commercial Pt/C, Au_2_Ag NCs, and AuAg_12_ NPs, respectively. As a result, among all tested catalysts the Au_3_Ag NFs demonstrated the best catalytic stability of MOR.

Further to meet the requirements for practical fuel cell applications, all catalysts were cycled between 0 and 1.6 V at a sweeping rate of 20 mVs^−1^ in 2 M methanol and 0.5 M KOH for a duration of 500 cycles to evaluate the long-term MOR performance (Supplementary Fig. 15). The mass activities of the Au_3_Ag NFs and Au_2_Ag NCs, respectively increased from 894.5 to 950.0 mA mg^−1^ and 136.1 to 141.4 mA mg^−1^ after 50 cycles, reflecting an initial activation process. After 100 cycles, the corresponding MA values dropped to 79 and 72%, and further to 12 and 15% after 300 cycles. By contrast, the mass activity of the commercial Pt/C decreased substantially from 590.9 to 271.1 mA mg^−^^1^ (54% loss) after only 50 cycles, and further to 58.4 mA mg^−1^ (90% loss) after 100 cycles. As for the AuAg_12_ NPs, The MOR performance is very poor with a negligible current density after 300 cycles. Noteworthy, there was no apparent change in the structure of Au_3_Ag NFs after the first 100 cycles, but some fractured and conglobated species start to occur after the extended 500 cycles (Supplementary Fig. 16). More importantly, the initial stepped surfaces tend to be flattened out showing less defect sites due to the rearrangement of surface atoms, which might account for the observed decay in MOR performance (Supplementary Fig. 17). Collectively, the above CV and chronoamperometry tests clearly demonstrate the superior MOR activity and stability of Au_3_Ag NFs against Au_2_Ag NCs and AuAg_12_ NPs, as well as the commercial Pt/C.

### CO-promoted MOR activities

Given that CO poisoning is one of the vital issues in devastating the MOR stability, both CO stripping and purging experiments were carried out to interrogate the impact of CO adsorption on the catalytic behavior of all catalysts. Supplementary Fig. 9 shows the CO stripping curves of all Au–Ag nanostructures in 0.1 M HClO_4_, where both the CO oxidation potentials of Au_3_Ag NFs and Au_2_Ag NCs at 0.59 and 0.73 V are far below their MOR peak–current potentials at 0.92 and 1.15 V, respectively. No much difference between the CV curves before and after CO stripping was observed for AuAg_12_ NPs, indicating Au are the main CO absorption site. The shoulder peak at 0.5 V overlapped with CO oxidation on Au_3_Ag NFs and diminished after CO stripping is likely due to the CO-induced OH^−^ adsorption on Au^34^. By contrast, Pt/C exhibits a CO oxidation peak at 0.92 V, much higher than its methanol oxidation potential at 0.78 V (Supplementary Fig. 9d). This result provides direct evidence for the poor MOR stability of Pt/C by CO poisoning, corroborating the previous *I*_f_/*I*_b_ comparison. Furthermore, by integrating the area of CO stripping and subtracting the area of Ag oxidation, the electroactive surface area can be obtained for Au_3_Ag NFs, Au_2_Ag NCs, AuAg_12_ NPs, and Pt/C being 27.8, 19.2, 4.9, and 68.89 m^2^ g^−1^, respectively. These values were used in calculating the specific activities due to the similar oxidation nature of MOR and CO stripping.

CO was purged into the electrolyte containing 2 M methanol to examine the evolution of MOR kinetics on the catalysts (Fig. 4e, f). Surprisingly, upon CO bubbling the peak current density of MOR on Au_3_Ag NFs increased abruptly from 950.0 to 1339.8 mA mg^−1^, followed by further gradual increment to 1438.6 mA mg^−1^ in the next 10 cycles. After cutting off the CO supply, the peak current density dropped back to 1113.5 mA mg^−1^ by the end of subsequent 10 cycles, but still higher than the original value without CO purge (Fig. 4e). As a comparison, the peak current of MOR catalyzed by Pt/C decreased from 530.1 to 239.4 mA mg^−1^ after purging CO for 10 CV cycles, and kept declining even when CO was shut off (Fig. 4f). This set of experiments clearly illustrate while CO is poisonous for Pt, it actually promotes the MOR on Au_3_Ag NFs. Similar CO-promoting behavior on the Au (111) surface was also observed by Rodriguez et al., who attributed the enhanced reactivity to the enhancement in electrostatic OH^−^ bonding, as a result of the change in electrostatic surface potential (work function) when CO is adsorbed atop the gold surface^34,57,58^. We note that these observations are conceptually analogous to the homogeneous electrocatalysis catalyzed by organometallics, of which the catalytic properties of the active metal center can be tuned by tailoring its coordination with organic ligands, such as the Monsanto and Cativa processes using CO as the ligand^59,60^. In our case of Au_3_Ag NFs we also surmise the adsorption of CO promotes the anionic OH^−^ binding to the alloyed surface, which in turn effectively catalyzes the deprotonation of methanol. This argument is further supported by the lowered potentials of OH^−^ adsorption and Au oxidation when comparing the Au_3_Ag NFs with other catalysts in CV measurements under alkaline conditions (Supplementary Fig. 18).

**Fig. 5:**
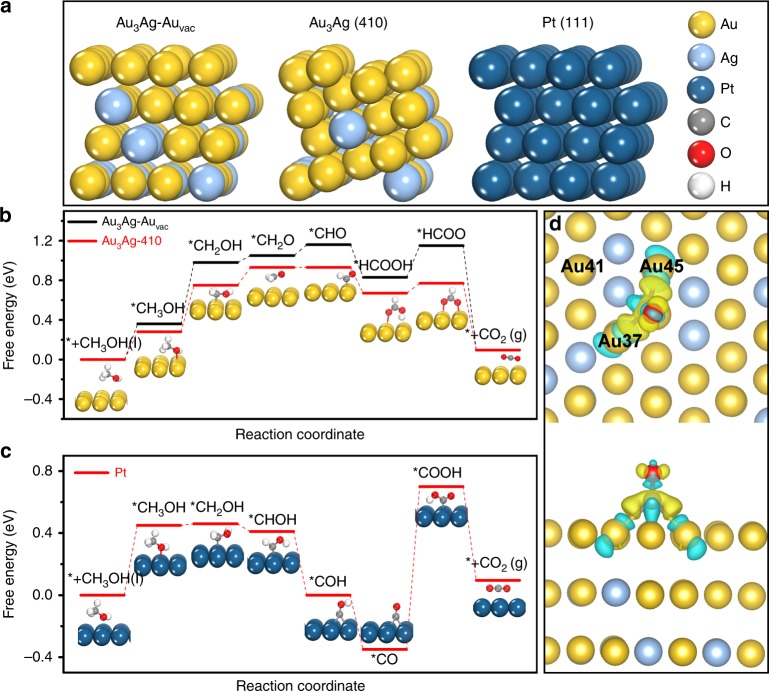
Simulated methanol oxidation mechanisms on the catalyst surfaces. **a** Stable configuration of Au_3_Ag–Au_vac_, Au_3_Ag (410), and Pt (111) surface. **b**, **c** Calculated free energy change of methanol oxidation intermediates on the Au_3_Ag–Au_vac_, Au_3_Ag (410), and Pt (111) surfaces, insets are the corresponding configurations of adsorbed intermediates. **d** Differential charge density of adsorbed CO on Au_3_Ag–Au_vac_, where the yellow and cyan contour with an iso-surface value of 0.0001 e/bohr^3^ indicate reduced and enhanced charge density, respectively. The H, O, C, Au, Ag, and Pt atoms are in white, red, grey, yellow, light blue, and dark blue colors, respectively

To further elucidate the defect-mediated activity enhancement on Au_3_Ag NFs and the CO-promoting effect, we applied the first-principles method in conjunction with the computational hydrogen electrode model to investigate the reaction free energy and activation barrier of all elementary steps along the reaction coordinate (see Supporting information for calculation details). As the structure of Au_3_Ag NFs is highly complex comprising a myriad of defects, in view of a reasonable computational cost we mainly considered two of the most abundant defect types representing the highly unsaturated surface, namely the high-index facets and atomic vacancies. Figure 5a and Supplementary Fig. 19a illustrate the constructed Au_3_Ag (410) and Au_3_Ag–Au_vac_ surface models, in comparison to the Au_3_Ag (111), Au (111), and Pt (111) references. The methanol adsorption on these surfaces takes similar configuration with the CH_3_OH molecule residing atop the Au (Pt) atom via the O bonding (Supplementary Fig. 20). Notably, the surfaces of Au_3_Ag (410) and Au_3_Ag–Au_vac_ possess more negative adsorption energies (−0.18 and −0.13 eV, respectively) than those of the Au_3_Ag (111) (−0.09 eV), Au (111) (−0.10 eV), and Pt (111) (−0.05 eV), implying the defect-mediated high-methanol affinity of the Au_3_Ag NFs surfaces (Supplementary Table 3).

Then, on the surfaces of Au_3_Ag (410), Au_3_Ag–Au_vac_, Au_3_Ag (111), Au (111), and Pt (111) all possible elementary steps of MOR were considered involving both deprotonation and hydroxyl addition. The various routes of reaction cascade are shown in Supplementary Fig. 21 and the optimized intermediates are displayed in Supplementary Fig. 20. The Au_3_Ag (111) and Au (111) surfaces share the same reaction cascade with very similar free energy change of each elementary step and the same potential-determined step (pds) of *CH_3_OH → *CH_2_OH (Supplementary Fig. 19b). The pds free energy change for Au_3_Ag (111) and Au (111) are 0.80 and 0.79 eV (Supplementary Table 5), respectively, suggesting that the introduction of Ag would not improve the intrinsic MOR activity of Au (111). However, after adopting crystalline defects such as the high-index facet of Au_3_Ag (410) and atomic vacancies of Au_3_Ag–Au_vac_, an alternate non-CO pathway (e.g., CH_3_OH(l) → *CH_3_OH → *CH_2_OH → *CH_2_O → *CHO → *HCOOH → *HCOO → CO_2_(g)) is taken and the adsorption free energies of all the intermediates are reduced, especially on the Au_3_Ag (410) surface (Fig. 5b). Note that the pds is still *CH_3_OH → *CH_2_OH but with a much reduced free energy change of 0.62 and 0.47 eV on the Au_3_Ag–Au_vac_ and Au_3_Ag (410) surfaces, respectively (Supplementary Table 5). This result strongly corroborates the experimental observations that the introduction of high-index atomic steps and crystalline defects can immensely lower the free energy barrier of pds, leading to greatly enhanced MOR activity.

On the Pt (111) surface, the reaction cascade follows the sequence CH_3_OH (l) → *CH_3_OH → *CH_2_OH → *CHOH → *COH → *CO → *COOH → CO_2_ (g), with the further reduced adsorption free energy of *CH_2_OH and the most negative free energy of *CO (Fig. 5c). Thus, the pds is relocated to the elementary step of *CO → COOH with an ultrahigh free energy change of 1.06 eV, in comparison to the pds (*CH_3_OH → *CH_2_OH) of 0.80 eV on Au_3_Ag (111), 0.62 eV on Au_3_Ag–Au_vac_ and 0.47 eV on Au_3_Ag (410). The observation of strong intermediates binding on Pt (111) with the highest *CO activation barrier well explains its high activity but poor stability. Collectively, the above computational evidences suggest that introducing crystalline defects and low-coordination atoms onto the Au–Ag surface enables to modulate the MOR reaction pathway, the intermediates binding energetics, and the rate-determining steps, ultimately leading to enhanced MOR activity and stability.

Using the transition state search method corrected with working potentials relative to the reversible hydrogen electrode (RHE), we further calculated the activation barrier of each elementary step for Pt (111) and Au_3_Ag-Au_vac_ in account of the high computational cost of such calculations. The results are shown in Supplementary Fig. 22-25 and listed in Supplementary Tables 6 and 7. On the Au_3_Ag–Au_vac_ surface, the rate-determining step (rds) is *CH_3_OH → *CH_2_OH with an activation barrier of 0.52 eV, which is also lower than that of Pt (111) with 0.58 eV for the rds *CO → *COOH. These kinetic studies are in good agreement with the above discussions on thermodynamics, and both are in line with the observed enhancement in MOR activity on Au_3_Ag NFs, rationalized by the low-coordination Au atoms on surface in mediating the intermediates binding and lowering the activation barriers. It is worth to mention the actual surface of Au_3_Ag NFs is much more complicated than these simple and exemplary defect models by encompassing rich steps, vacancies, dislocations, and grain boundaries, which should further promote its intrinsic activity. In fact, according to the Brønsted–Evans–Polanyi correlation^61^, the lower reaction free energy change of the elementary steps on Au_3_Ag (410) (Fig. 5b) might result in lower activation barriers than those observed on the Au_3_Ag–Au_vac_ surface.

Lastly, to further interrogate the CO-promoted OH^−^ adsorption for the facilitation of subsequent deprotonation, we calculated the charge distribution of adjacent Au atoms after CO adsorption on the surface of Au_3_Ag–Au_vac_ through the differential charge density and bader charge analysis (Fig. 5d). The Au atoms around the adsorbed CO molecule such as Au37, Au45, and Au41 all contribute partial electron densities to CO, inducing the polarized Au^δ+^ with ΔC_Au41_ = 0.0027, ΔC_Au45_ = 0.0567, and ΔC_Au37_ = 0.0464. These positively charged Au^δ+^ atoms are conducive to OH^−^ adsorption through Coulomb interactions, which in turn catalyzes the methanol β-hydrogen deprotonation^34^. As one can see from Fig. 5b and Supplementary Fig. 19b, the step of β-hydrogen deprotonation (i.e., *CH_3_OH → *CH_2_OH) is the pds for all Au-based catalysts investigated in the current study. Therefore, the introduction of CO not only does not poison the Au_3_Ag NFs catalyst, but instead promotes the MOR activity.

## Discussion

Taken together, the superior MOR activity of Au_3_Ag NFs can be mainly ascribed to the highly exposed active sites on the open 3D framework, and the bimetallic Au–Ag alloying for generating abundant defects on the ultrathin edges and vertices, including low-coordination atoms, vacancies, dislocations, stacking faults, and lattice strains, etc. These structural defects enable to actively interact with the absorbents (both the methanol molecules and reaction intermediates) owing to the enriched surface electronic states and shifted d bands, which is evident from the XPS and XAFS analysis. In the d-band model proposed by Nørskov and co-workers^62^, the adsorption of rate-limiting intermediates is related to the electronic structure of the catalyst, where the valence p orbitals of the absorbents and intermediates form bonding and antibonding states with the metal d-band^63–65^. When the metal coordination number is low, the d-band width becomes narrower and the d-band center shifts up toward the Fermi level, pushing more of the antibonding states above *E*_f_, resulting in decreased occupation and more active interaction with the absorbents. The shift of d bands is further enhanced by the adsorption of oxygen-containing species, which on the surface of Au_3_Ag NFs is synergistically promoted by lowered oxidation potentials and CO co-absorption. It has been reported that CO-promoted OH^−^ adsorption on gold efficiently catalyzes the beta-hydrogen elimination from CH_3_OH, which is the rate-limiting step in typical Au-catalyzed MOR^66,67^. We also note that not only the Au defects but also the Ag atoms on Au_3_Ag NFs might conjunctively facilitate the OH^−^ adsorption. Moreover, the positively polarized Au^δ+^ sites have been reportedly shown enhanced Au oxidation activities^68^. As a synergy of all the above factors, the electrokinetics of MOR is greatly enhanced through the mediation of intermediate binding and corresponding activation barriers.

To summarize, in this work bimetallic Au_3_Ag nanoframes composed of ultrathin edges and vertices were synthesized using a facile one-pot method with high structural uniformity. By employing a comprehensive suite of structural and compositional characterization techniques, the morphological evolution and formation mechanism of these nanoframes were well elucidated. CS-TEM revealed rich crystalline defects on the nanoframes, resulting in modulated electronic structure with characteristic Au^δ+^ polarization and prominent shift of d bands. Elaborative voltammetry measurements unveiled higher electrochemical activity of the defect-rich Au_3_Ag nanoframes in OH^−^ adsorption, surface oxidation, as well as CO stripping. Consequently, Au_3_Ag NFs demonstrated extraordinary MOR activities with an onset potential of only 0.3 V and a high SA of 3.38 mA cm^−^^2^, 3.9 times that of the commercial Pt/C. The superior MOR of Au_3_Ag NFs is mainly attributed to the highly exposed active sites, abundant surface defects, and modulated electronic structure, effectively mediating the reaction pathway, intermediates binding, and rate-determining steps for improved MOR electrokinetics. What is more, MOR catalyzed by the Au_3_Ag NFs has been shown counter-intuitively promoted by CO owing to the much lowered CO-stripping potential and synergistic CO–OH^−^ interaction on the catalyst surface. Through the tailoring of Au nanostructure into defect-rich frameworks, this study signifies the approach by mediating surface electronic states to impart unprecedented catalytic activities.

## Methods

### Chemicals

Silver nitrate (AgNO_3_, ≥99.0%), cuprous chloride (CuCl, ≥98.0%), octadecylamine (ODA, ≥90%), and cetyltrimethylammonium bromide (CTAB, ≥99.0%) are purchased from Shanghai Aladdin Bio-Chem Technology Co., Ltd. Chloroauric acid tetrahydrate (HAuCl_4_·4H_2_O, ≥47.8% Au basis), ethanol (C_2_H_6_O, ≥99.7%), cyclohexane (C_6_H_12_, ≥99.0%), dichloromethane (CH_2_Cl_2_ ≥ 99.5%), methanol (CH_4_O, ≥99.5%), and potassium hydroxide (KOH, ≥99.0%) are purchased from Sinopharm Chemical Reagent Co., Ltd. All chemicals were used without further purification. Milli-Q water (>18.0 MΩ cm) was purified with a Sartorius arium mini ultrapure water system.

### Synthesis of Au_3_Ag nanoframes

Totally, 0.073 g CTAB were dissolved in 10 ml ODA which kept at 125 °C under nitrogen (N_2_) for 1 h. Then 0.02 g AgNO_3_ and 0.01 g CuCl were added into this system. After 36 min of magnetically stirring, 0.01 g HAuCl_4_·4H_2_O were quickly injected, and the solution was kept for another 10 min. When the reaction was finished, the reaction flask was carefully transferred into a water bath of 60 °C in order to quench the growth process, followed by addition of 40 mL ethanol and kept for 6 h without magnetic stirring. The produced Au_3_Ag nanoframe was further washed 15 times with ethanol and dichloromethane mixed solution, and redispersed in cyclohexane.

### Characterization

The crystal structure was characterized by powder X-ray diffraction (XRD, Bruker AXS D8 Advance diffractometer with Cu Kα source). The surface morphology and microstructure were observed using a dual-beam electron microscope (SEM, FEI Scios) and a field-emission transmission electron microscope (TEM, FEI TECNAI G2 F20 200 kV) equipped with an energy-dispersive X-ray analyzer (EDX). Spherical-aberration-corrected TEM (Cs-corrected TEM, FEI Titan Themis Cubed G2 300) was used to inspect atomic orientation of edges and vertexes in Au_3_Ag nanoframe. Surface elements were probed by X-ray photoelectron spectroscopy (XPS, Thermo Fisher, Escalab 250Xi) using a monochromatic Al Ka (1486.6 eV) X-ray source, with all binding-energy values calibrated with C 1s ¼ 284.6 eV. Inductively coupled plasma optical emission spectroscopy (ICP-OES) was utilized for total composition analysis with PerkinElmer Optima 8000. The Au L_3_-edge XAFS spectra were recorded by QEXAFS at Beamline 44A at the Taiwan Photon Source (TPS) and transmission mode were applied to all of the samples. The raw data and the fittings were carried out by using IFEFFIT software packages according to the standard analysis procedures^69^.

### Electrochemical characterization

In all, 0.1 mg AuAg nanostructures was suspended in a solution containing 0.5 ml ethanol, 40 ul of 5 wt% Nafion solution and 5 ug Ketjen Black by ultrasonication for 30–40 min to complete the loading process. To fabricate working electrode, a quantity of 50 ul of the suspension was drop-casted on glassy carbon (GC) electrode surface (loading amount was about 0.5 mg/cm^2^). The c was air dried for 1 h at room temperature before use. All the electrochemical measurements were performed in a standard three-electrode cell at room temperature using an electrochemical workstation (CHI660E). The Pt wire was used as the counter electrode, with the Ag/AgCl electrode (filled with saturated KCl) as the reference electrode. Prior to catalyst loading, the GC electrode was carefully polished with 1.0, 0.3 and 0.05 μm alumina powder in sequence, and cleaned by sonication in ethanol and deionized water. The electrolyte was 0.5 M KOH and 2 M methanol (PH = 13.3) bubbled with nitrogen for 30 min prior to MOR measurements. All the potentials were converted to the potentials referring to the RHE, according to E (vs. RHE) = E (vs. Ag/AgCl) + 0.059 pH + 0.198.

### Estimation of electrochemical surface area

CO stripping was used for determination of the electrochemical surface area. First, the catalyst was subjected to an electrochemical cleaning procedure by cycling the electrode in N_2_ purged 0.1 M HClO_4_ for 30 cycles in a potential window of −0.2 to 1.5 V at a scan rate of 100 mV s^−1^. Then CO (>99.99% purity) gas was bubbled in the electrolyte for 30 min while holding the electrode potential at −0.1 V to allow for the adsorption of a monolayer of CO molecules. Afterward, dissolved CO was removed by bubbling the electrolyte for 20 min with N_2_ gas. Finally, the voltammogram for CO stripping was recorded in a potential window of −0.2 to 1.5 V for two consecutive cycles at a scan rate of 20 mV s^−1^. The first cycle is to record the CO_ads_ stripping and the next cycle to ensure the complete stripping of CO_ads_ during the first cycle.

$${\mathrm{ECSA}} = \frac{{Q_{\mathrm{CO}}}}{{Q_0 \times m}}{.}$$

Where *Q*_CO_ is the integrated charge consumed during the CO_ads_ oxidation, *Q*_0_ is the standard charge required for oxidation of a CO_ads_ monolayer on a Pt surface with a value of 420 µC cm^−2^, and Au with 450 µC cm^−2^. *m* was the Au or Pt mass on the working electrode as determined by ICP-OES

## Supplementary information

Supplementary Info-No highlight

## Data Availability

The data that support the findings of this study are available from the authors upon request.
