# Supplementary material for: Octahedral gold-silver nanoframes with rich crystalline defects for efficient methanol oxidation manifesting a CO-promoting effect
Source: Nat Commun. 2019 Aug 22;10:3782. doi: 10.1038/s41467-019-11766-w (PMC6706449; doi:10.1038/s41467-019-11766-w)
Supplement: Supplementary file 1 — Supplementary Info-No highlight [file 41467_2019_11766_MOESM1_ESM.pdf]

Supplementary Information

Octahedral Gold-Silver Nanoframes with Rich Crystalline Defects for Efficient Methanol Oxidation Manifesting a CO-promoting Effect

Xiong et al

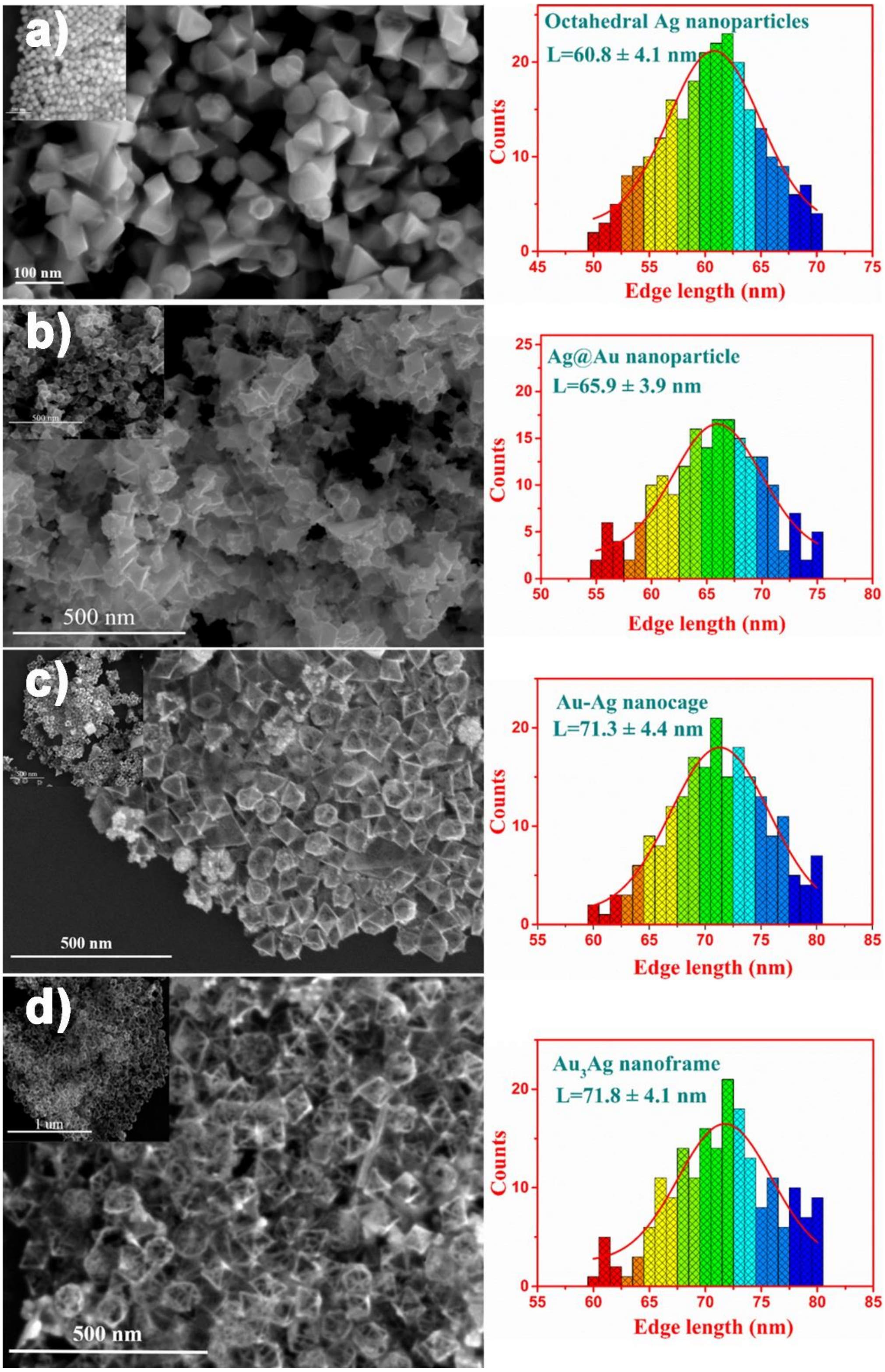

Supplementary Figure 1. SEM images and particle size statistics of Au-Ag nanostructures. **a** Octahedral Ag nanoparticles, **b** Ag-Au nanoparticles, **c** Au-Ag nanocages, **d** Au-Ag nanoframes.

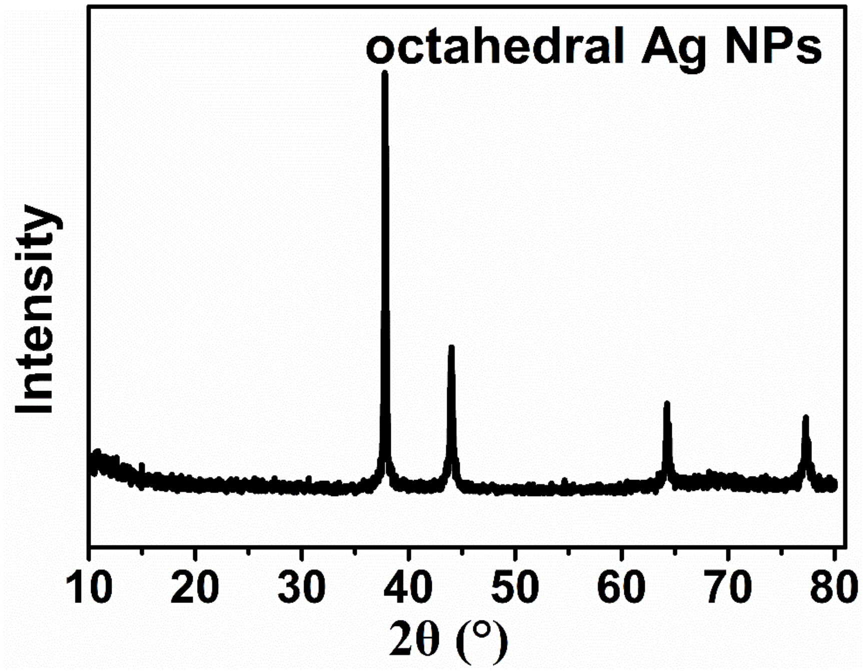

Supplementary Figure 2. XRD spectra of the octahedral Ag nanoparticles.

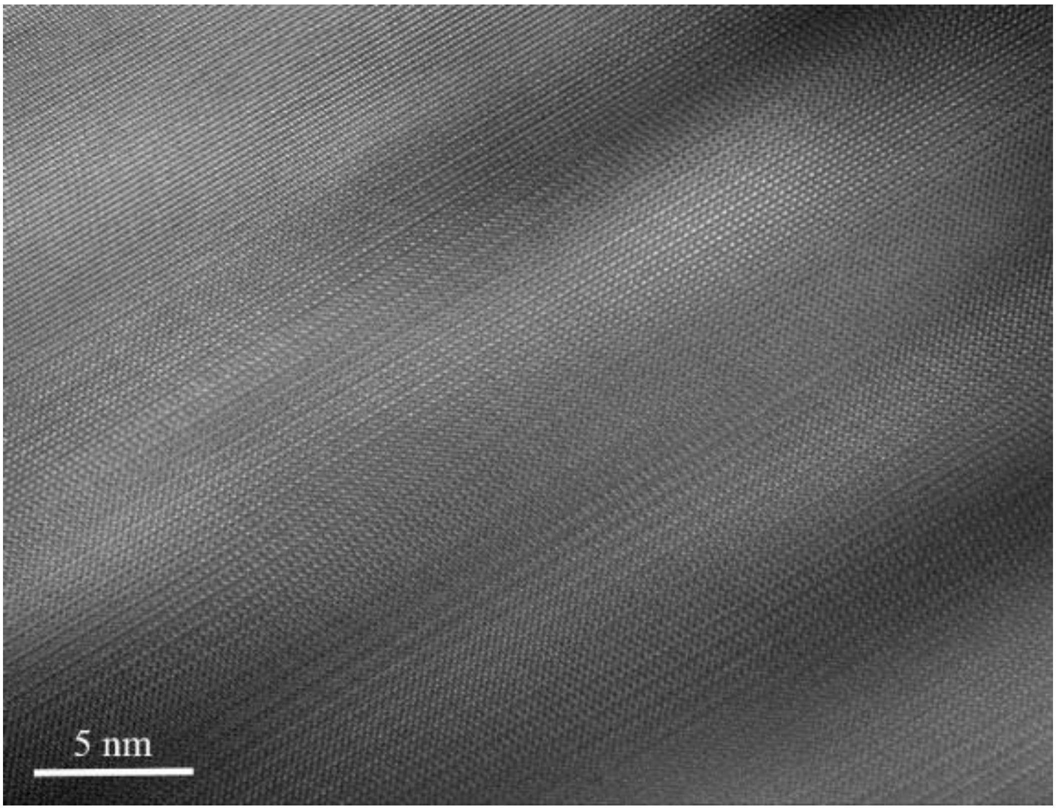

Supplementary Figure 3. Cs-TEM image of the octahedral Ag nanoparticles, showing perfect lattice structure without obvious defects.

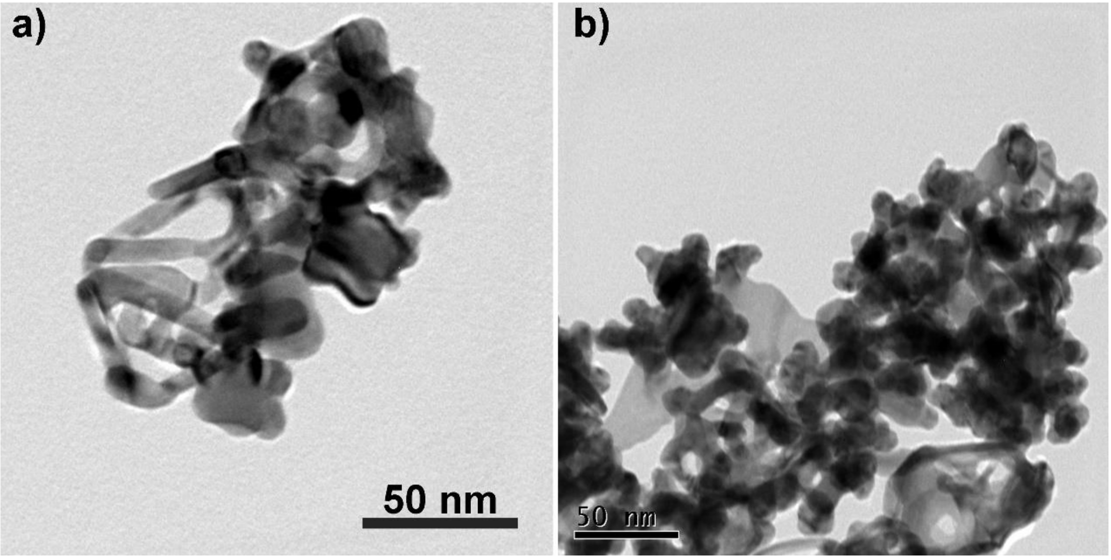

Supplementary Figure 4. TEM images of over-reacted samples obtained from different reaction time. **a** and **b** Products were obtained at the 60th minute.

Supplementary Table 1 | Composition analysis of Ag-Au nanoparticless, Au-Ag nanocages and Au-Ag nanoframe by EDX, XPS and ICP.

Method	Au:Ag atomic ratio		
	**Ag-Au Nanoparticles**	**Au-Ag nanocages**	**Au-Ag nanoframes**
**EDX**	**8:92**	**71:29**	**78:22**
**XPS**	**7:93**	**68:32**	**72:28**
**ICP**	**1:12.4**	**2.1:1**	**3.3:1**

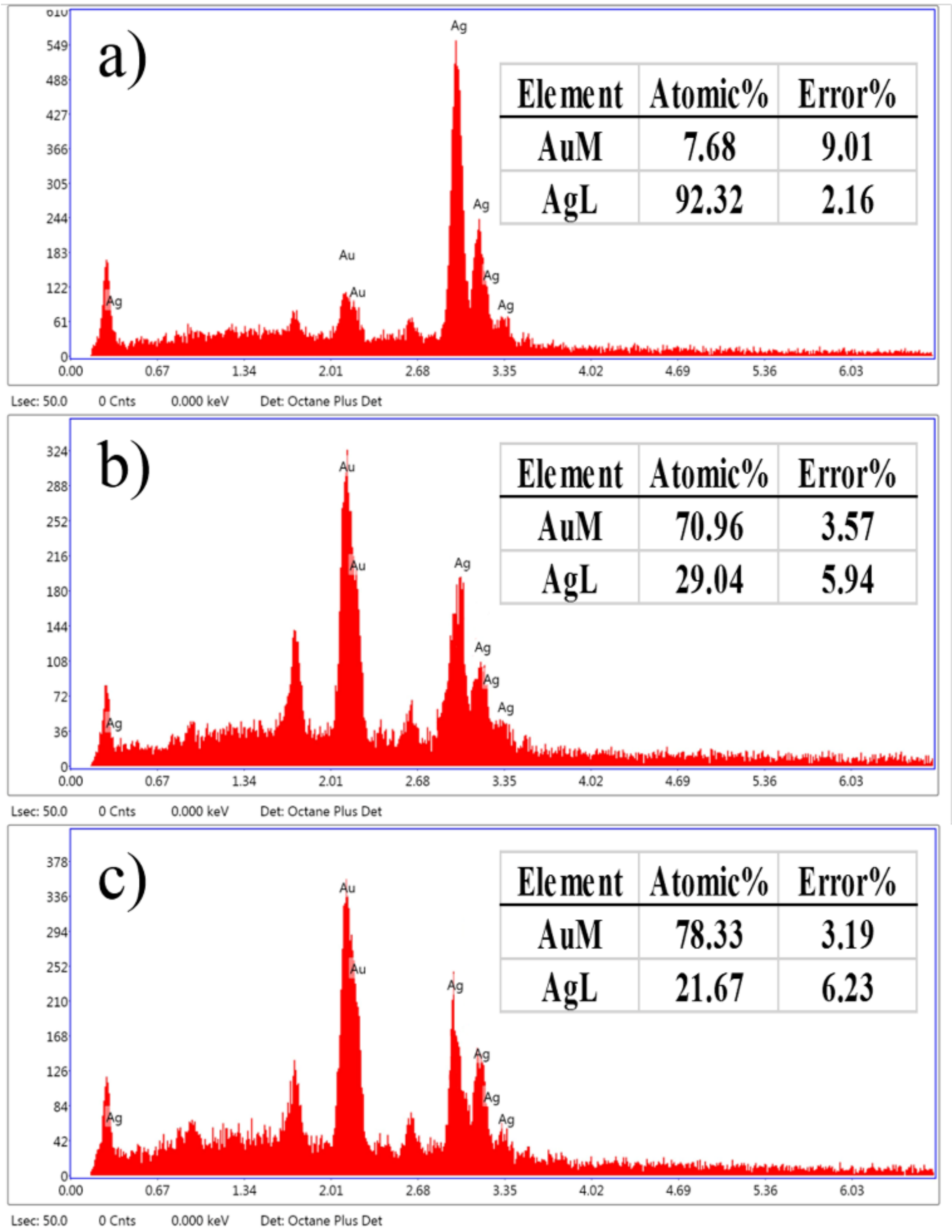

Supplementary Figure 5. EDX analysis of the Au-Ag nanostructures. The Si signal is from the substrate. **a** Ag-Au nanoparticles, **b** Au-Ag nanocages, **c** Au-Ag nanoframes.

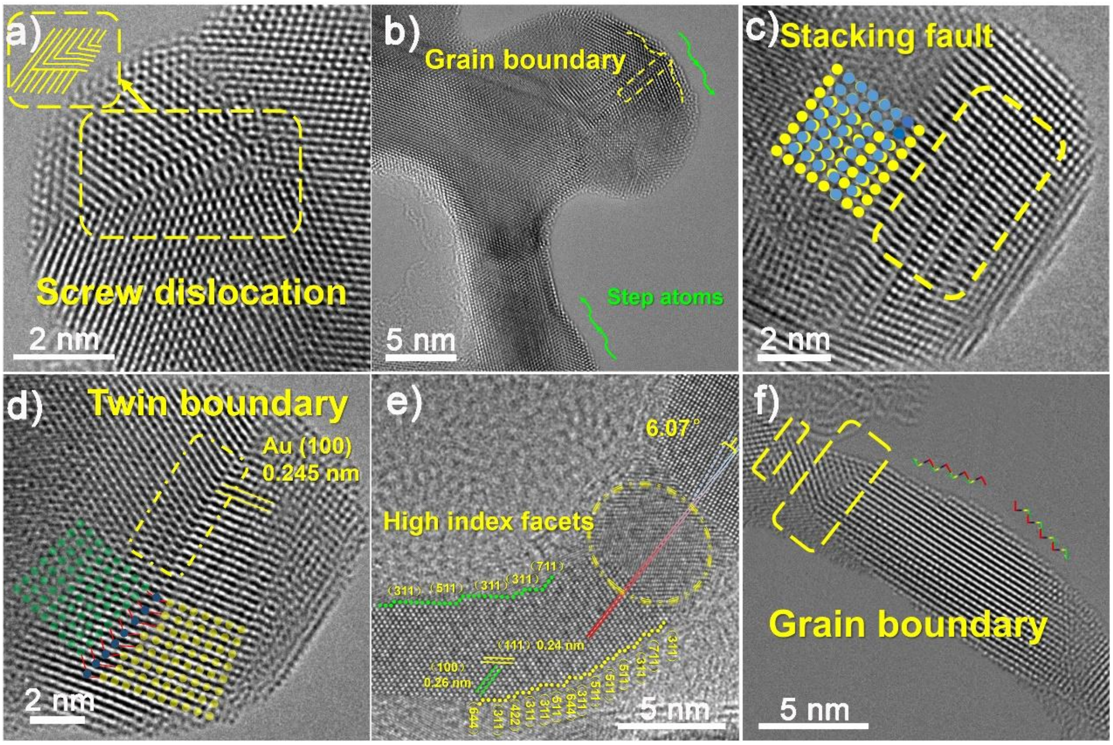

Supplementary Figure 6. Cs-TEM images of additional sections of Au_3Ag NFs, showing abundant defects in the edges and vertices. **a** the yellow-dotted box highlights a screw dislocation in the vertex region with its atomic arrangement schematically depicted by the yellow lines shown in the top-left corner. The screw dislocations provide self-perpetuating steps to enable the anisotropic growth of various nanomaterials at low supersaturation. **b** the yellow-dotted box shows an obvious grain boundary, and the green lines mark the surface of the nanoframe is packed with stepped atoms. **c** A stacking fault parallel to the Au (111) surface is remarked by the yellow-dotted box. In the modeled diagram illustrating the Au atomic alignment in this dislocation area, the blue balls represent the atoms above the yellow ones. **d** the yellow-dotted box illustrates a twin boundary in the vertex with the corresponding diagram of atomic arrangement shown below. **e-f** edges of the nanoframe full of stepped surfaces and grain boundaries.

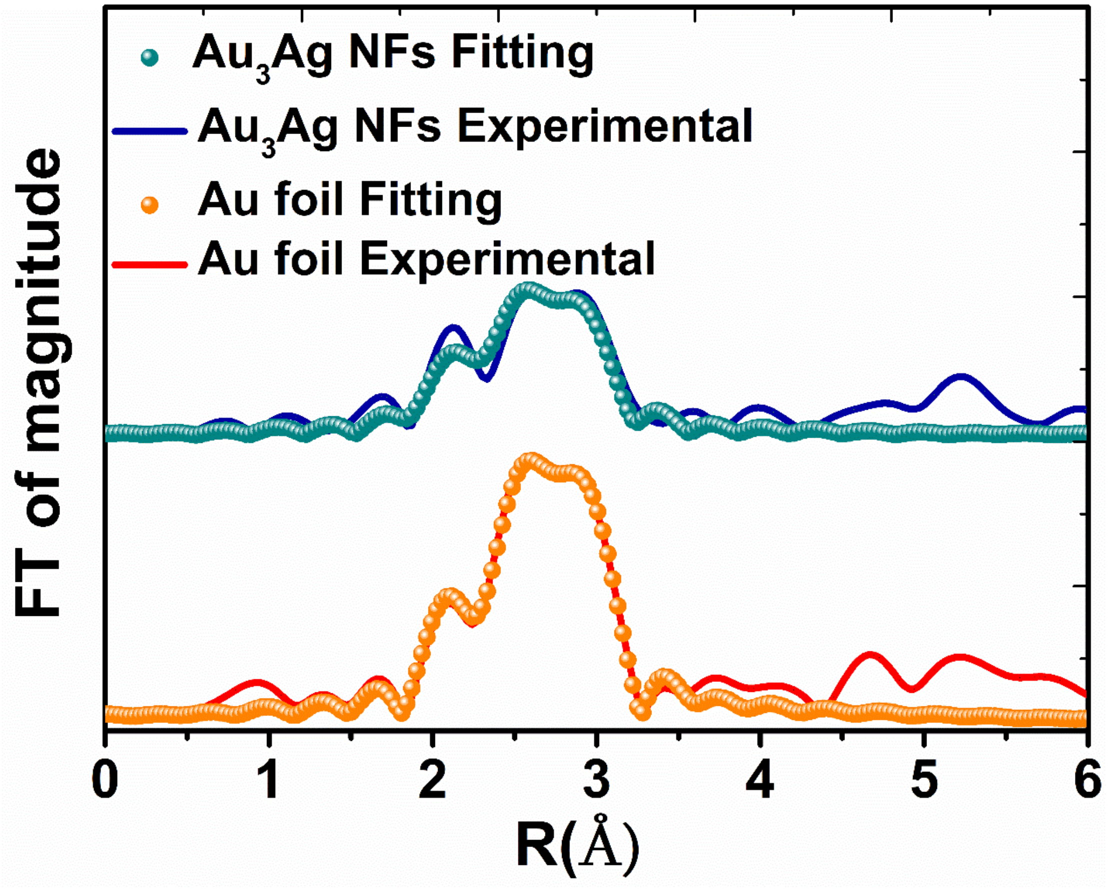

Supplementary Figure 7. FT-EXAFS spectra obtained from Au L_3-edge x-ray absorption near-edge structure of the Au foil and Au_3Ag NFs

Supplementary Table 2 | Structural parameters of Au_3Ag NFs from the Au L_3-edge EXAFS analysis

**Sample**	Bond Type	CN[a]	R(Å)[b]	σ^2 (Å^2)[c]
**Au foil**	**Au-Au**	**12**	**2.86**	**0.008**
**Au_3Ag NFs**	**Au-Au**	**9.4**	**2.84**	**0.01**

[a]CN means coordination number. [b]Error: R ≤ ± 0.01Å. [c]Debye-Waller factors. Error: σ^2 ≤ ± 0.0004 Å.

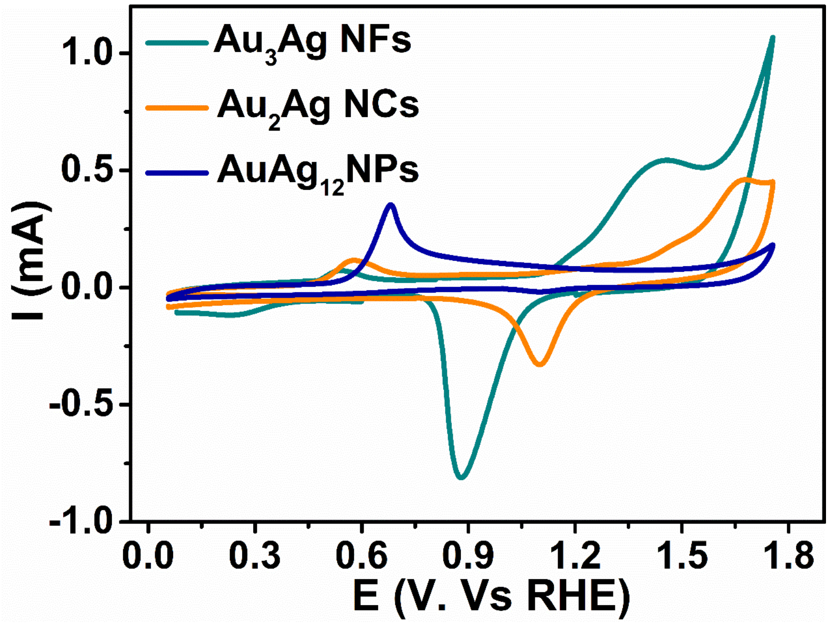

Supplementary Figure 8. Cyclic voltammograms of the Au-Ag nanostructures in 0.1M $HClO_4$ electrolyte at a potential sweep rate of 20 mV s^{-1}. The Au_3Ag NFs shows earlier Ag and Au oxidation potentials than Au_2Ag NCs and $AuAg_{12}$ NPs.

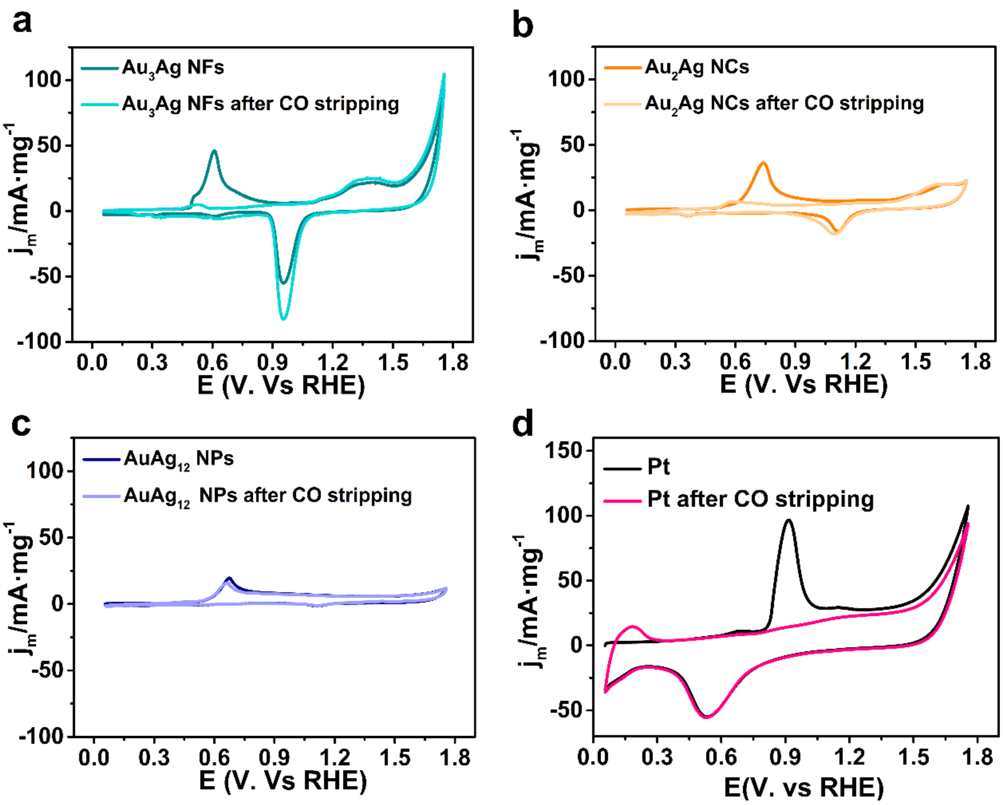

Supplementary Figure 9. The CO-stripping curves of **a** Au_3Ag NFs, **b** Au_2Ag NCs, **c** $AuAg_{12}$ NPs and **d** Pt/C in 0.1 M $HClO_4$.

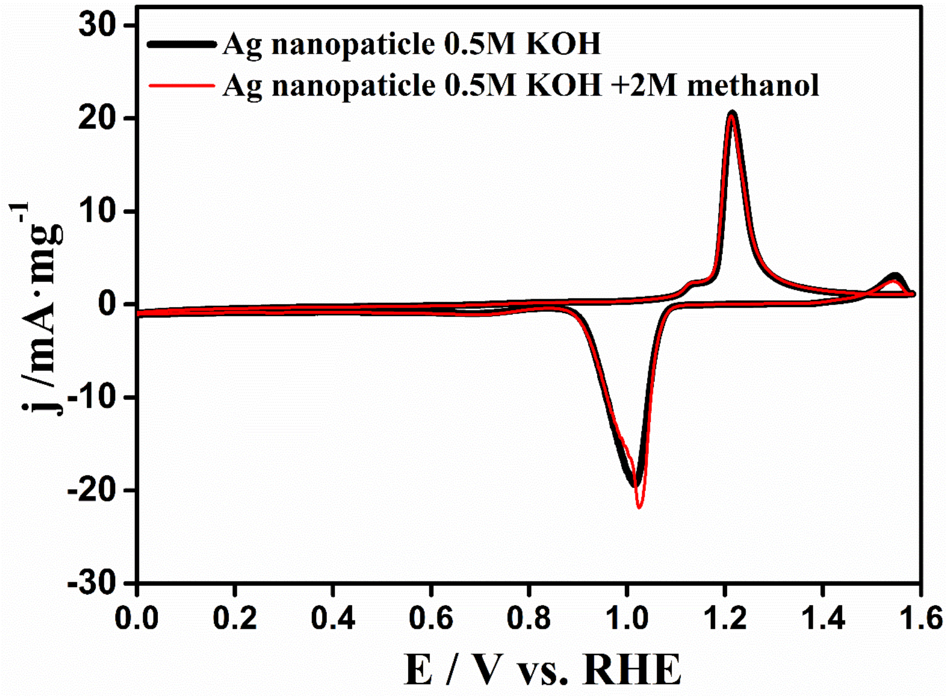

Supplementary Figure 10. Cyclic voltammograms of the octahedral Ag nanoparticles in 0.5 M KOH with 2 M methanol or without methanol.

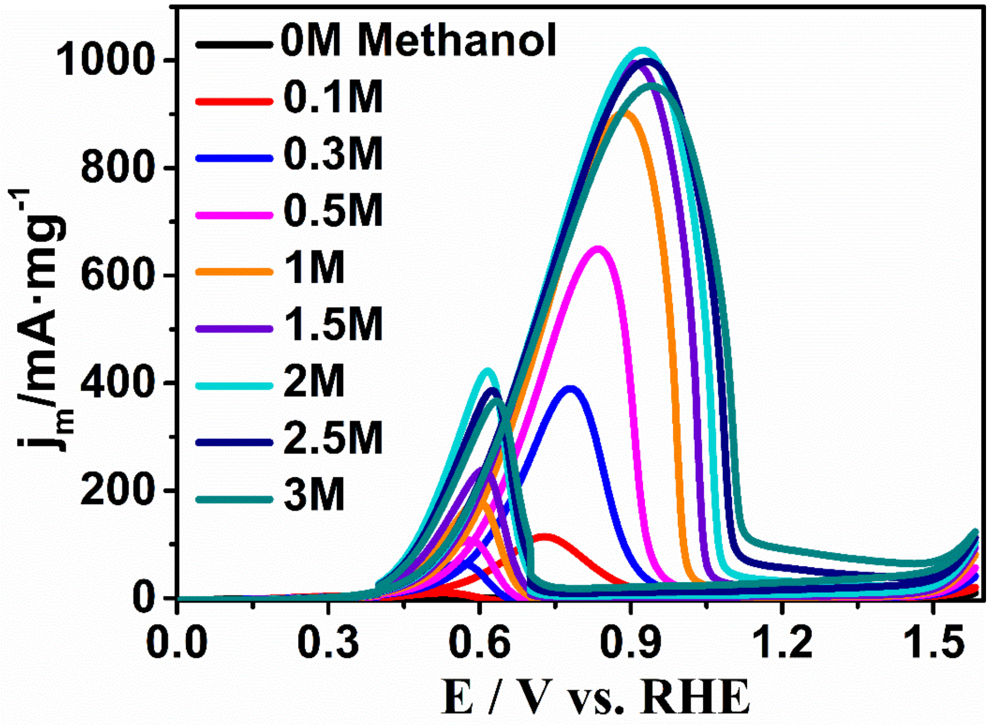

Supplementary Figure 11. Cyclic voltammograms of Au_3Ag NFs in 0.5 M KOH with different concentrations of CH_3OH at a sweep rate of 20 mV s^{-1}

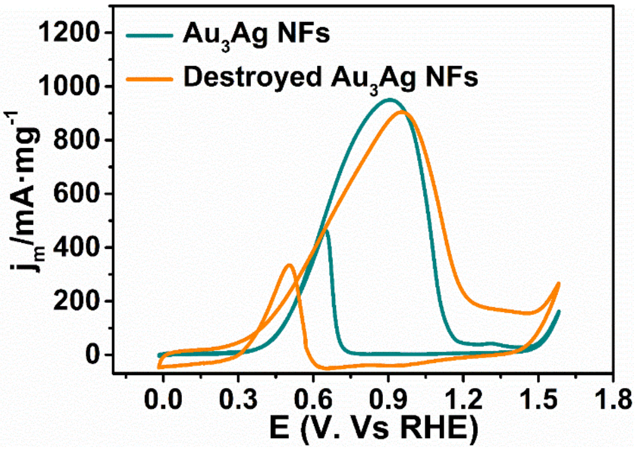

Supplementary Figure 12. Electrochemical MOR performance of Au_3Ag NFs before and after ball milling in deoxygenated solutions of 2 M CH_3OH and 0.5M KOH taken at a sweep rate of 20 mV s^{-1}.

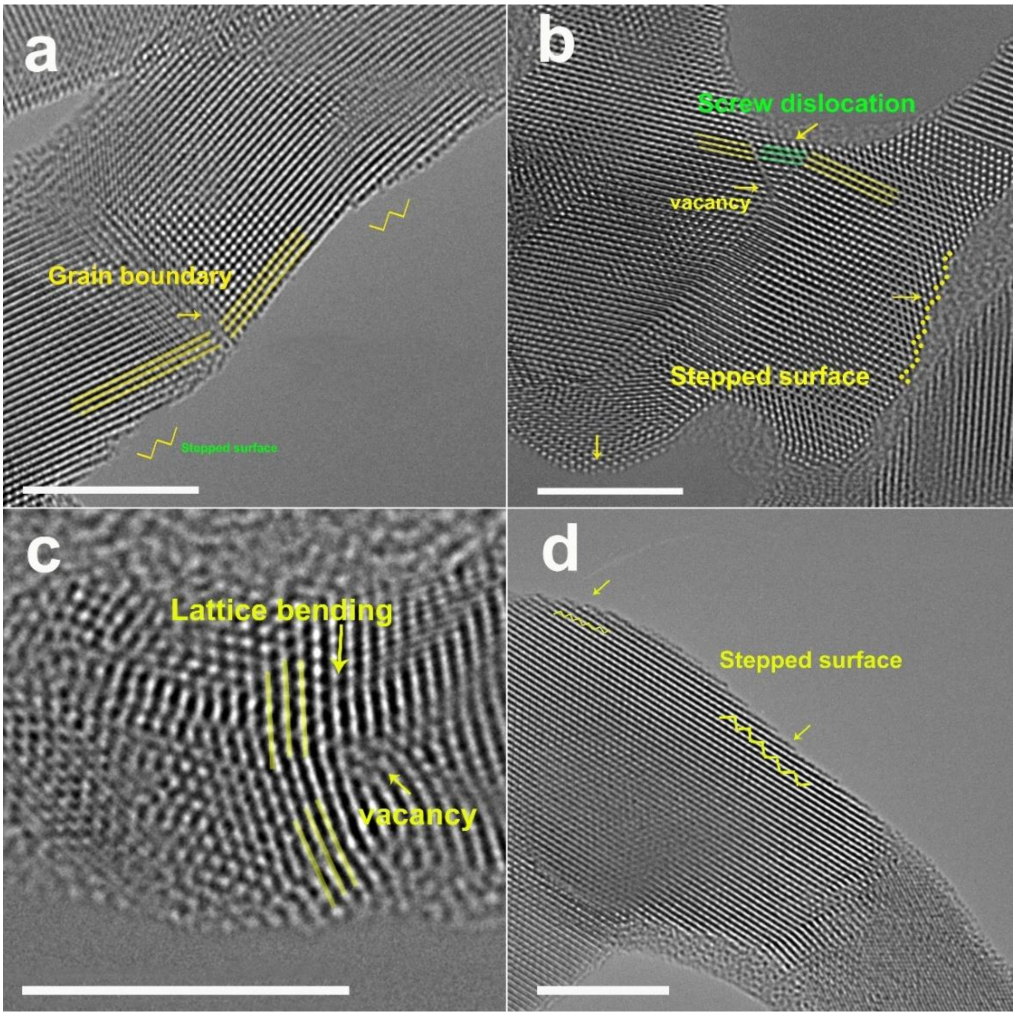

Supplementary Figure 13. Cs-TEM images of destroyed Au_3Ag NFs by ball milling, showing mostly unaffected crystalline defects.

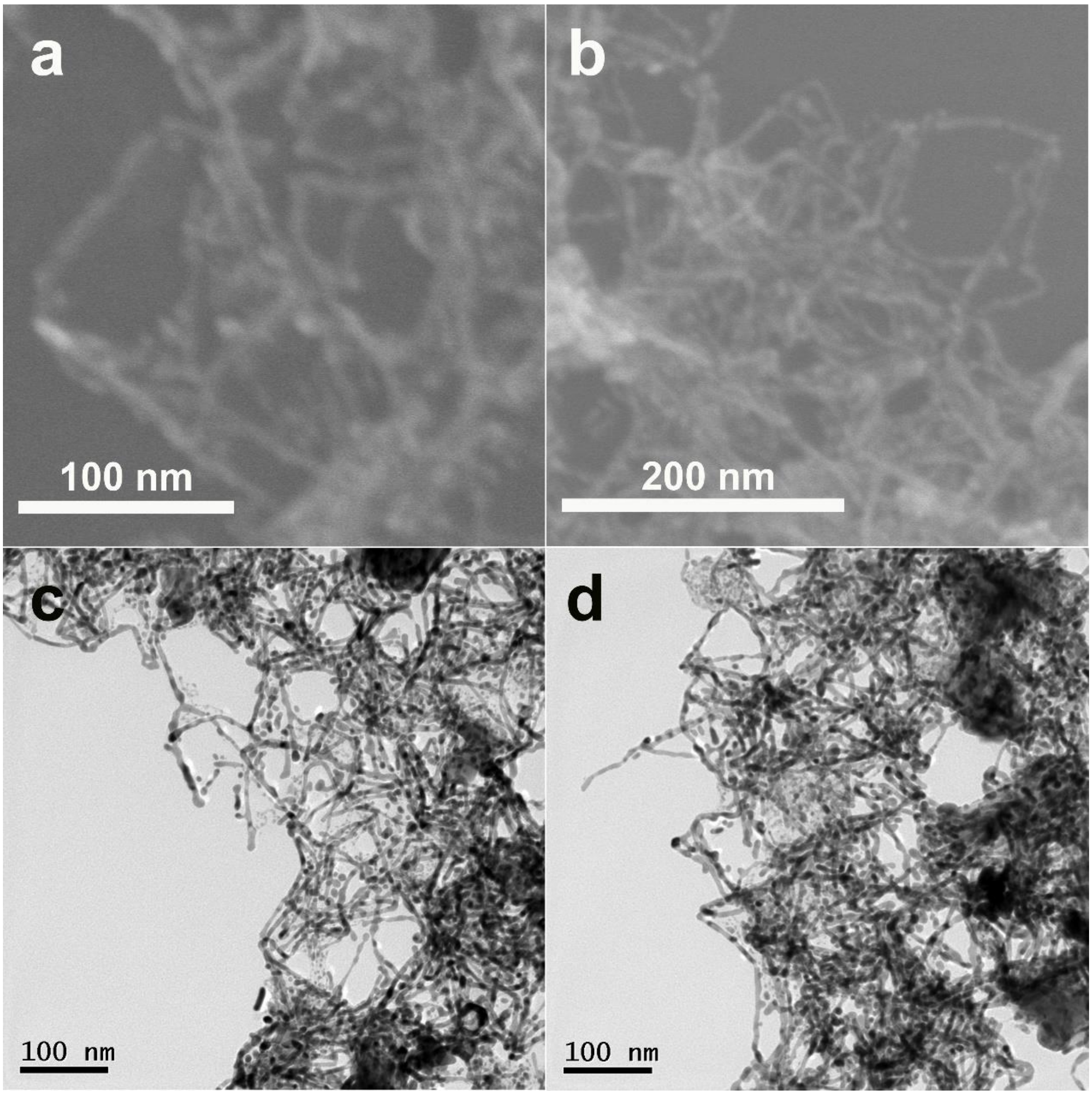

Supplementary Figure 14. SEM (**a**, **b**) and TEM images (**c**, **d**) of the destroyed Au_3Ag NFs by ball milling.

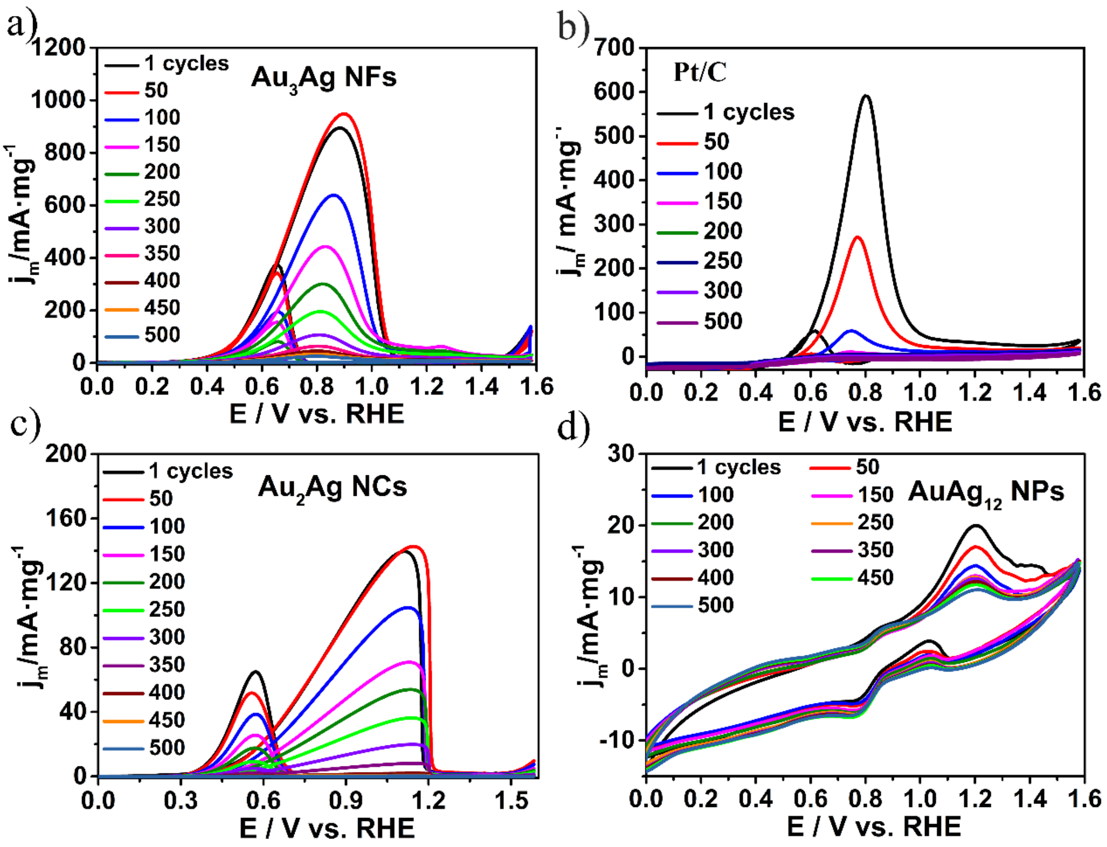

Supplementary Figure 15. Electrochemical durability of different catalyst samples inspected by repeated CV cycling in 0.5 M KOH and 2M methanol at a sweep rate of 20 mV s^{-1}. **a** Au_3Ag NFs, **b** Commercial Pt/C, **c** Au_2Ag NCs and **d** $AuAg_{12}$ NPs.

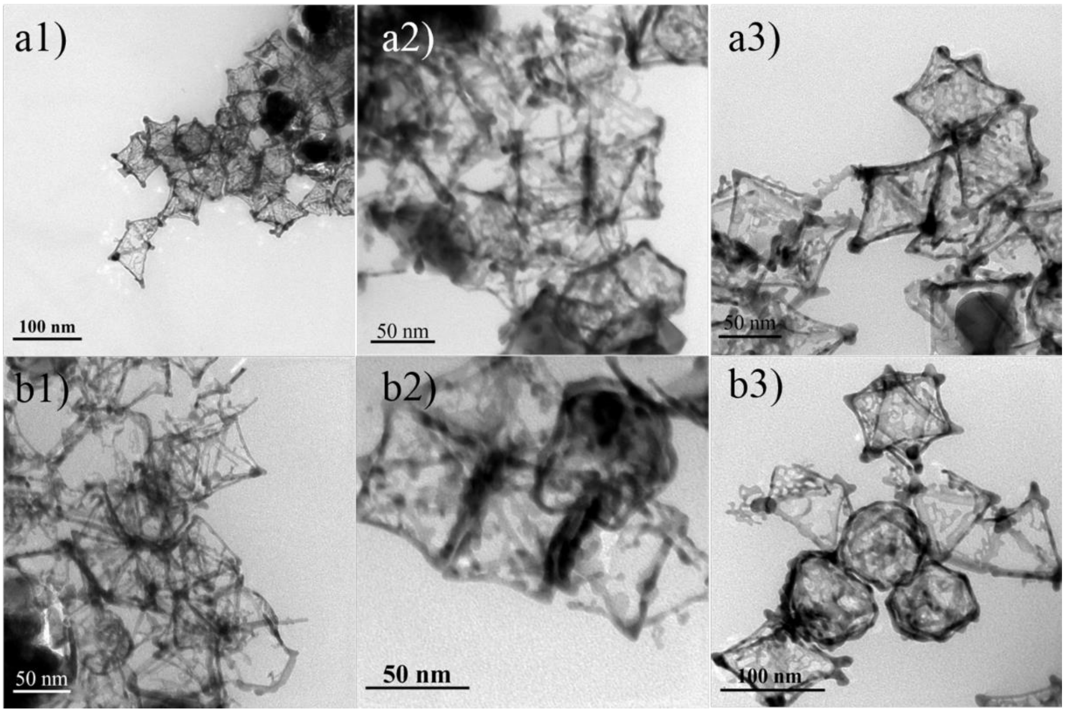

Supplementary Figure 16. TEM images of Au_3Ag NFs after various CV cycles in 0.5 M KOH and 2 M CH_3OH. (**a1 - a3**) after 100 cycles, and (**b1 - b3**) after 500 cycles.

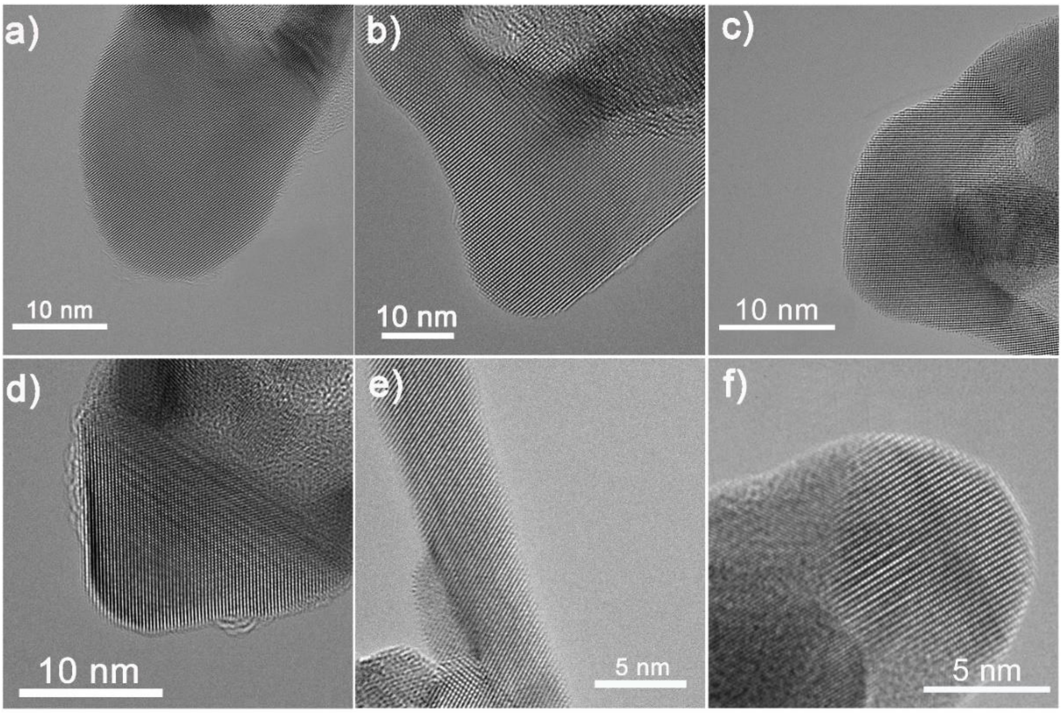

Supplementary Figure 17. Cs-TEM images of Au_3Ag NFs after 500 MOR CV cycles, showing the loss of stepped atoms and lattice defects due to atomic surface rearrangement on both the edges and vertexes.

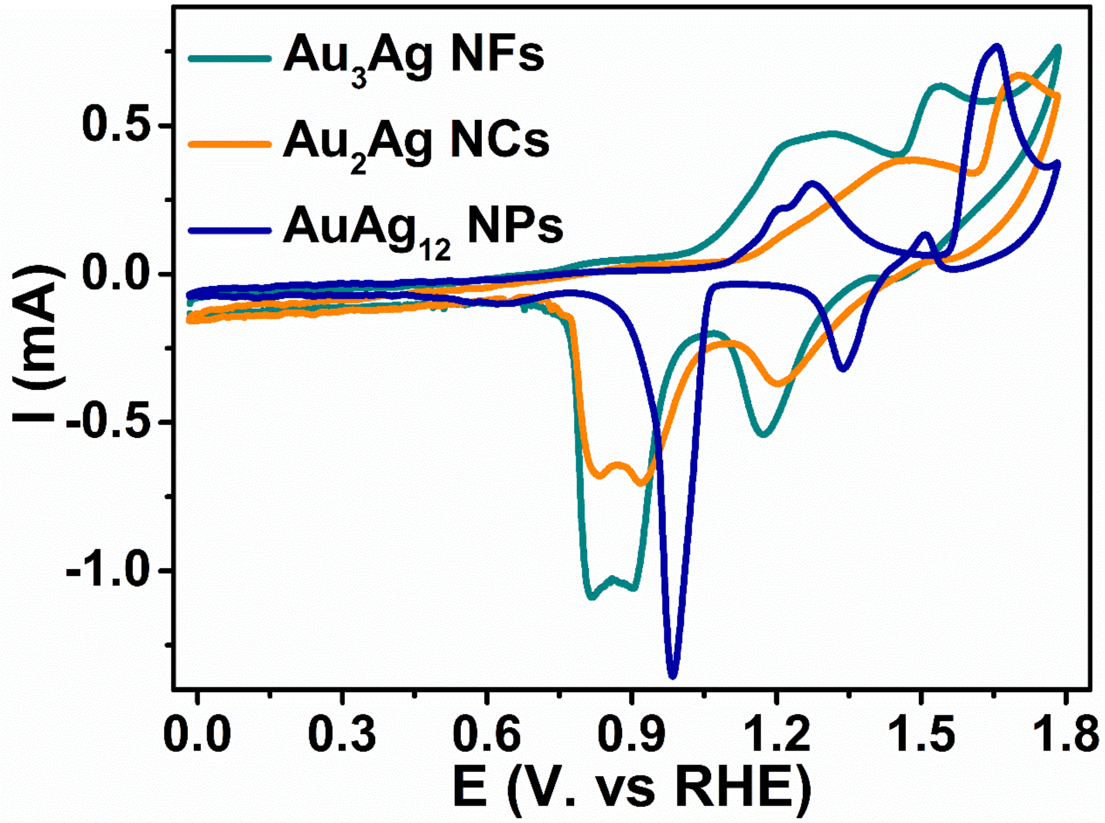

Supplementary Figure 18. Cyclic voltammograms of the Au-Ag nanostructures in 0.5 M KOH at a potential sweep rate of 20 mV s^{-1}.

Supplementary Note 1

To examine the electrochemical behaviors of the Au-Ag nanostructures in alkaline conditions which are applied in the MOR studies, CV scans were taken in 0.5M KOH. Comparing to those acquired in $HClO_4$ (**Supplementary Fig. 18**), the voltammograms obtained here are more complicated as they involve a multi-step oxidation for both Au and Ag (eq. 1 – eq. 4), with the current peaks superimposed with each other. Taking the Au_3Ag NFs as an example, the oxidation peaks emerge from ~0.80 V, followed by two prominent peaks at ~1.21 and 1.53 V, in accordance with the surface OH^- adsorption, the oxidation of Ag to Ag_2O and AgO, and the Au oxidation[1]. On the cathodic sweep, three reduction peaks are prominent at 1.17, 0.90 and 0.82 V, corresponding to the reduction of AgO, Ag_2O and AuO, coupled with the desorption of OH^-.

$$2Ag + 2OH^- \longrightarrow Ag_2O + H_2O + 2e^- \quad (1)$$

$$2Ag_2O + 2OH^- \longrightarrow 2AgO + H_2O + 2e^- \quad (2)$$

$$Au + OH^- \longrightarrow Au\text{-}OH_{ads}^{(1-\lambda)^-} + \lambda e^- \quad (3)$$

$$Au\text{-}OH_{ads}^{(1-\lambda)^-} + OH^- \longrightarrow AuO + H_2O + (2-\lambda)e^- \quad (4)$$

By contrast, all oxidation peaks of Au_2Ag NCs occur at significantly higher potentials than those of Au_3Ag NFs, suggesting a lower surface activity. In addition, when comparing Au_2Ag NCs with Au_3Ag NFs, the convoluted AuO/Ag_2O reduction peaks at 0.82 - 0.9 V indicate a higher coverage of oxidized species on the Au_3Ag NFs on account of their larger electroactive surface area. As for $AuAg_{12}$ NPs, all major peaks on both anodic and cathodic sweeps are mainly relevant to the oxidation and reduction of AgO and Ag_2O, except for the small reduction peak at 0.62 V, which is likely due to the reduction of AuO/Au-OH owing to the low Au content in $AuAg_{12}$ NPs. These observations provide additional evidence for the predominant Au occupation at the surface of Au_3Ag NFs with rich low coordination states and high chemical activities.

Supplementary Note 2

All calculations are carried out on the density functional theory (DFT), executed by the Vienna Ab-initio Simulation Package (VASP) code using the projector augmented wave (PAW) method to tackle electron-core interactions[2,3]. The exchange-correlation interactions are handled by generalized gradient approximation with the Perdew-Burke-Ernzerhof (PBE) function[4]. The kinetic energy cutoff with plane wave basis set is set to 400 eV. The first Brillouin zone integrations are adopted with $2 \times 3 \times 1$ k-points mesh by the Monkhorst-packing method[5]. We may also test higher accuracy simulations with the energy cutoff of 520 eV and $3 \times 4 \times 1$ k-points mesh to calculate the adsorption of CH_3OH molecule in Supplementary **Table 3.** The results of adsorption energy trend is similar to the 400 eV energy cutoff and $2 \times 3 \times 1$ k-points mesh. For the geometry optimization, all the structures were relaxed fully until the total energy and total force per atom is less than 2×10^{-5} eV and 0.02 eV/Å, respectively. The slabs exposed to (111) surface with $4 \times 2\sqrt{3}$ supercell and (410) surface with 3×1 supercell are cleaved from the bulk material and the thickness of the vacuum layer was about ~10 Å. The bottom two layers are fixed to the bulk position, and the other layers and adsorbates are fully relaxed. We use the Climbing Image-Nudged Elastic Band (CI-NEB)[6] method together with the Dimer method[7] to search for the transition state, which is just one imaginary frequency along the reaction pathway via the vibrational frequency calculations based on harmonic approximation.

The adsorption energy and bond distance of the CH_3OH molecule is calculated on

the Au (111), Au_3Ag (111), Au_3Ag (410), Au_3Ag-Au_{vac}, and Pt (111) surfaces as shown in Supplementary **Table 3** below. $E_{ads} = E_{total} - E_{surf} - E_{CH3OH}$, where E_{total} and E_{surf} are the total energy with and without CH_3OH, respectively. E_{CH3OH} is the molecule energy of CH_3OH. Note that the more negative the adsorption energy, the stronger the bonding.

In the electrochemical oxidation of CH_3OH, the elementary steps are referred to the pure Au and Pt surfaces as shown in Supplementary **Figure 21**, namely,

*A-H + OH^-_{aq}→*A + H_2O_{aq} + e^- (5)

where the reaction free energy changes can be expressed as: $\Delta G = G_{*A} + G_{H2O} + G_{e-} - G_{*A-H} - G_{OH-}$, $G_{OH^-} = G_{H2O} - G_{H+}$, and thus $\Delta G = G_{*A} - G_{*A-H} + G_{H+} + G_{e-}$. For the calculations of $G_{H+}+G_{e-}$ under the normal condition (pH=0, U=0), we employ the computational hydrogen electrode (CHE) model provided by Nørskov[8] to calculate the chemical potential of $H^+ + e^-$, $G_{H^+ + e^-} = 1/2G_{H2}$.

The reaction free energy correction was calculated according to the below formula: $\Delta G = \Delta E + \Delta ZPE + \Delta H$ (0→298.15K) - $T\Delta S$, where ΔE, ΔZPE, ΔH (0→298.15 K) and ΔS refer to the reaction energy from DFT calculations, the change of zero-point energy, the integrated heat capacity from 0 K to 298.15 K, and the entropy change, respectively. T of 298.15 K was used. The results are shown in Supplementary **Table 4.** The potential determined step (pds) is the maximum reaction free energy step among all elementary processes, the rate-limiting potential $U_{pds} = \Delta G/e$ V. Additionally, Potential-dependent reaction free energy $\Delta G(U) = \Delta G(0) - eU$, U is the working potential.

For the potential-dependent activation barriers, we refer to the previous work by Nie et al[9]. They have developed a universal method to simulate the potential-dependent activation barriers for elementary processes within the electrocatalytic reaction sequence using the non-electrochemical reaction. As known, electrochemical oxidation is the reverse reaction of electroreduction. Similarly, for the electrooxidation of surface-bound species, as shown in Eq. (5), we instead determine the activation barrier for an equivalent dehydrogenation reaction:

*A-H↔*A + *H (6)

This activation barrier, deduced from standard DFT calculations and zero-point vibrational energy (ZPE) correction, is labeled by E_a^0. The barrier is then assigned to the electrode potential at which the chemical potential of adsorbed *H is equal to the

chemical potential of H^++e^-. This potential is the equilibrium potential, U_0, for hydrogen desorption:

*A + *H↔*A + H^+ + e^- (7)

The value of U_0 is calculated using the equation $\Delta G = G_{*A} + (1/2G_{H2} - eU) - G_{*A} - G_{*H}$, by determining the potential at which the reaction free energy given by Eq. (7) is zero. Then the barrier is extrapolated to other electrode potentials using Butler-Volmer formalism[10]. As such, the potential-dependent (U) activation barrier ($E_a(U)$) is calculated below:

$$E_a(U) = E_a^0(U_0) - \beta'(U - U_0) \quad (8)$$

In Eq. (8), β' is an effective symmetric factor, which is approximated as

$$\beta' = \beta + (\mu_{TS} - \mu_{reactant})/d \quad (9)$$

Where β is a reaction symmetry factor between 0.3 and 0.7, with 0.5 indicating a symmetric reaction. We started with the approximation that β is 0.5 for all the elementary steps and used an approximation to correct β for an asymmetric reaction by considering variations in the interaction of surface dipole moment (μ) and interfacial electric field between the transition state and reactant state in Eq. (9). d is the the thickness of interfacial double layer, assumed as 3 Å.

Supplementary Table 3 | Adsorption energy and bond length between the Oxygen of CH_3OH and Au (Pt) atoms on the Au_3Ag (111), Au_3Ag (410), Au_3Ag-Au_{vac}, Au (111) and Pt (111) surfaces.

	Sample	Au_3Ag (111)	Au_3Ag-Au_{vac}	Au (111)	Pt (111)	Au_3Ag (410)
**520- 3 × 4 × 1**	E_{ads} (eV)	**-0.09**	**-0.13**	**-0.10**	**-0.05**	**-0.18**
	D_{M-O} (Å)	**2.85**	**2.69**	**2.78**	**2.37**	**2.58**
**400- 2 × 3 × 1**	E_{ads} (eV)	**-0.09**	**-0.12**	**-0.10**	**-0.03**	**-0.20**
	D_{M-O} (Å)	**3.03**	**2.67**	**2.87**	**2.39**	**2.59**

Supplementary Table 4 | ZPE, ΔH(0→298.15K) and TS of the free molecules and adsorbed intermediates (eV).

**molecule**	ZPE	ΔH(0→298.15K)	TS
**H_2**	**0.27**	**0.09**	**0.40**
**H_2O**	**0.55**	**0.10**	**0.67**
**CH_3OH**	**1.34**	**0.12**	**0.74**
**$*CH_3OH$**	**1.38**	**0.12**	**0.30**
**$*CH_2OH$**	**1.10**	**0.10**	**0.23**
***CHOH**	**0.79**	**0.08**	**0.16**
***CHO**	**0.46**	**0.07**	**0.15**
***CO**	**0.19**	**0.07**	**0.14**
***COOH**	**0.61**	**0.09**	**0.19**
**$*CH_3O$**	**1.08**	**0.10**	**0.20**
**$*CH_2O$**	**0.71**	**0.09**	**0.25**
***COH**	**0.49**	**0.07**	**0.12**
***HCOO**	**0.60**	**0.11**	**0.23**
***HCOOH**	**0.89**	**0.09**	**0.21**
***H**	**0.16**	**0.02**	**0.01**
***OH**	**0.35**	**0.11**	**0.05**

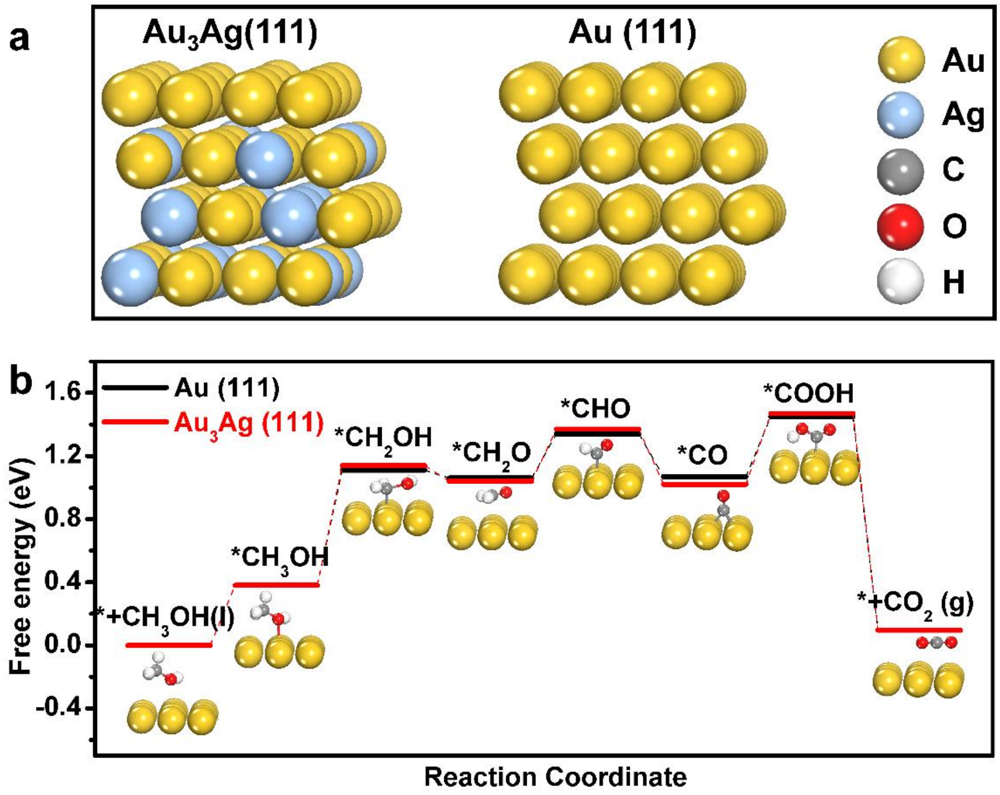

Supplementary Figure 19. a Atomic configurations of the Au_3Ag (111) and Au (111) surfaces. **b** Calculated free energy diagrams of MOR on the Au_3Ag and Au (111) surfaces. Insets are the corresponding intermediates configuration from the optimial reaction pathway. The H, O, C, Au, Ag and Pt atoms are in white, red, grey, yellow, light blue, and dark blue colors, respectively.

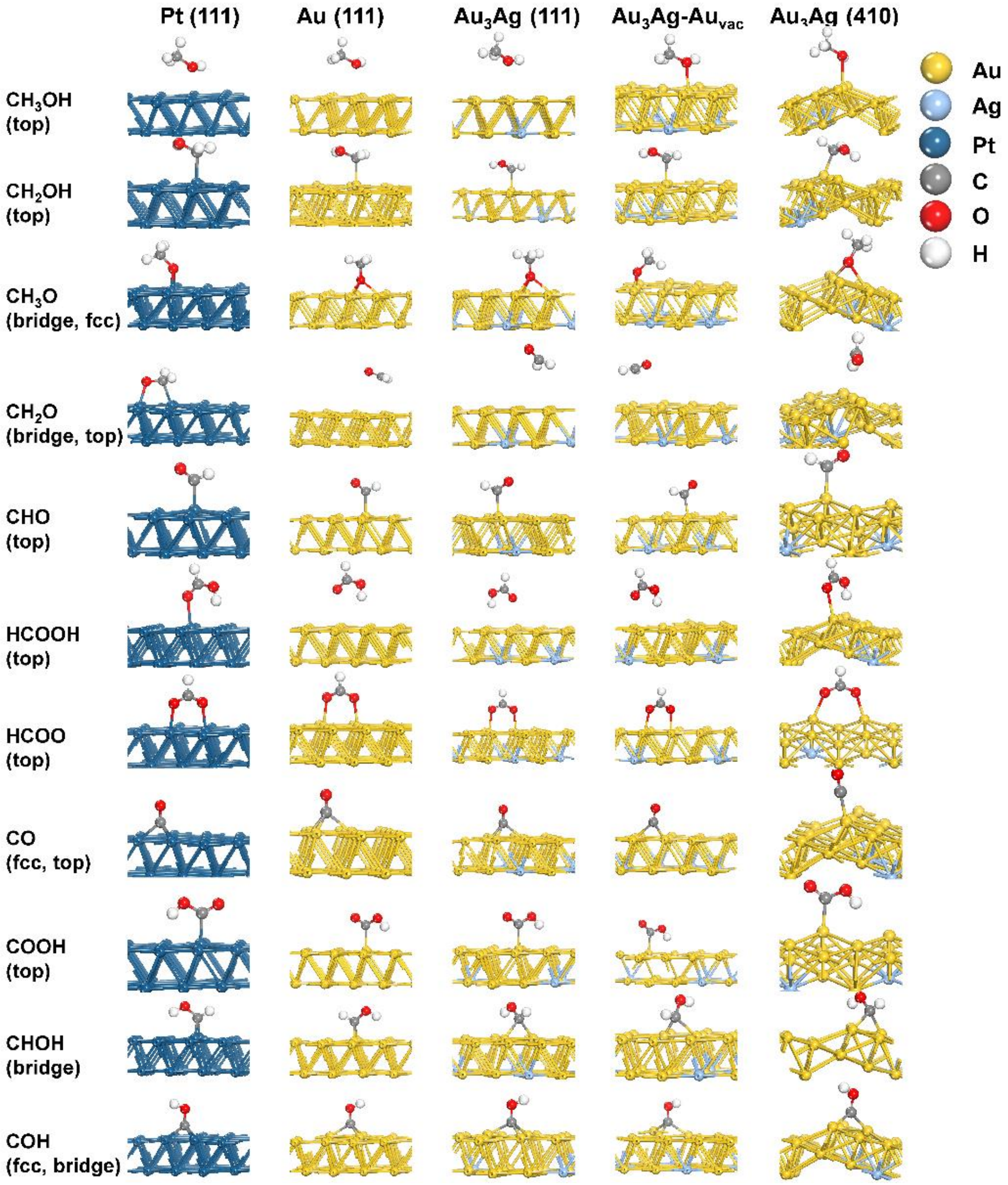

Supplementary Figure 20. Stable adsorption configurations of the MOR intermediates including CH_3OH, CH_2OH, CH_3O, CH_2O, CHO, HCOOH, HCOO, CO, COOH, CHOH, and COH from top down on the Pt (111), Au (111), Au_3Ag (111), Au_3Ag-Au_{vac}, and Au_3Ag (410) surface from left to right. The H, O, C, Au, Ag and Pt atoms are in white, red, grey, yellow, light blue, and dark blue color, respectively.

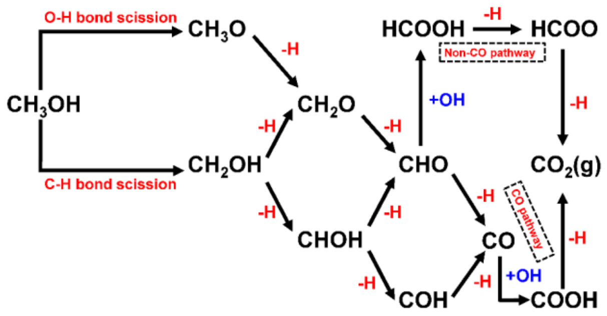

Supplementary Figure 21. CO pathway vs Non-CO pathway of Methanol Oxidation Reaction (MOR).

Supplementary Table 5 | Calculated free energy change of the MOR elementary steps on Pt (111), Au_3Ag-Au_{vac}, Au_3Ag (410), Ag_3Ag (111), and Au (111). Unit in eV.

Elementary step	Pt (111)	Au_3Ag-Au_{vac}	Au_3Ag (410)	Au_3Ag (111)	Au (111)
+CH_3OH(l)→CH_3OH	0.45	0.36	0.28	0.38	0.38
CH_3OH→CH_2OH	0.01	0.62	0.47	0.76	0.73
+CH_3OH(l) →CH_2OH	0.46	0.97	0.75	1.14	1.11
*CH_2OH→*CHOH	-0.05	0.35	0.61	0.68	0.72
*CHOH→*COH	-0.41	0.58	0.58	0.35	0.39
*CHOH→*CHO	0.04	-0.18	-0.42	-0.45	-0.49
*CHO→*HCOOH	0.22	-0.33	-0.26	-0.51	-0.46
*HCOOH→*HCOO	0.19	0.32	0.10	0.55	0.50
CH_2OH→CH_2O	0.53	0.07	0.18	-0.10	-0.05
*COH→*CO	-0.35	-1.27	-1.54	-1.16	-1.15
+CH_3OH (l)→CH_3O	1.33	1.23	1.02	1.62	1.61
CH_3OH (l)→CH_3O	0.88	0.87	0.74	1.24	1.23
CH_3O→CH_2O	-0.34	-0.19	-0.08	-0.58	-0.55
*CH_2O→*CHO	-0.54	0.11	0.00	0.33	0.28
*CHO→*CO	-0.80	-0.52	-0.54	-0.35	-0.27
*CO→*COOH	1.05	0.66	0.62	0.45	0.38

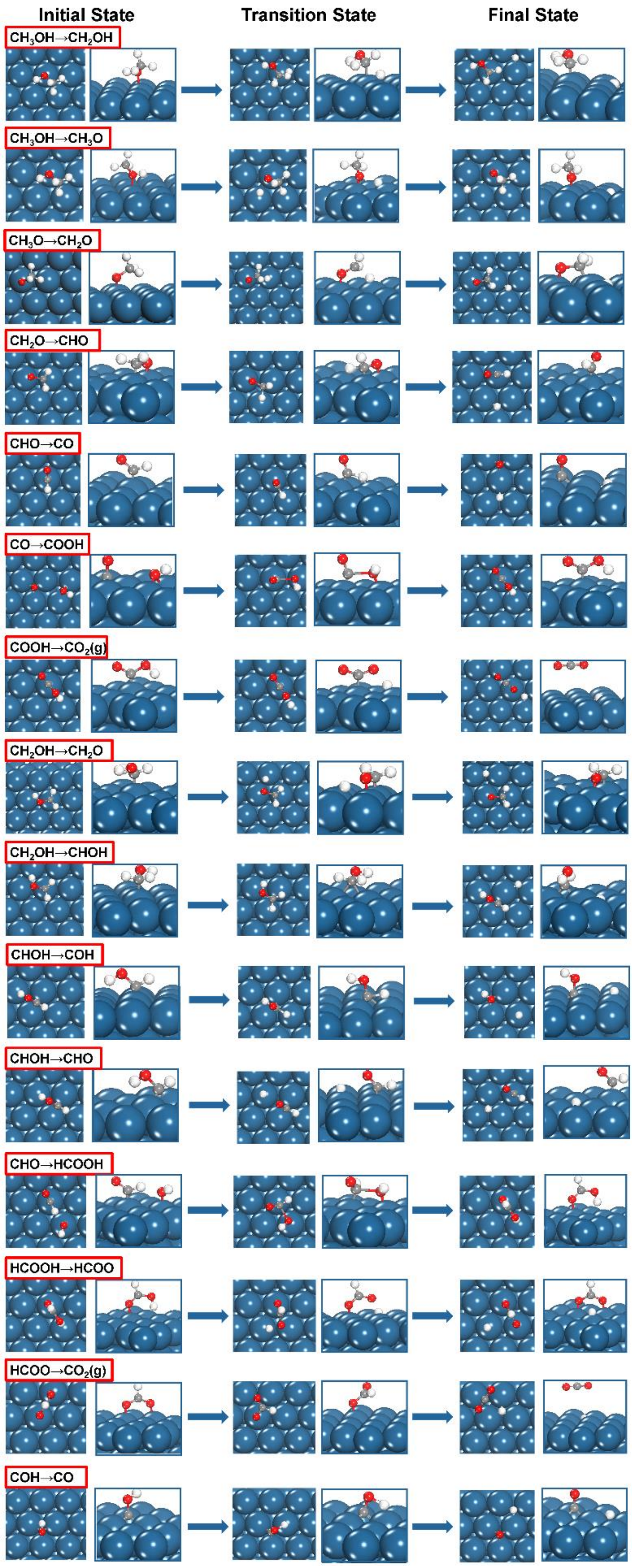

Supplementary Figure 22. Optimized geometries of the initial, transition, and final states of the MOR elementary steps on Pt (111). The Pt, C, O, and H atoms are in dark blue, grey, red, and white, respectively.

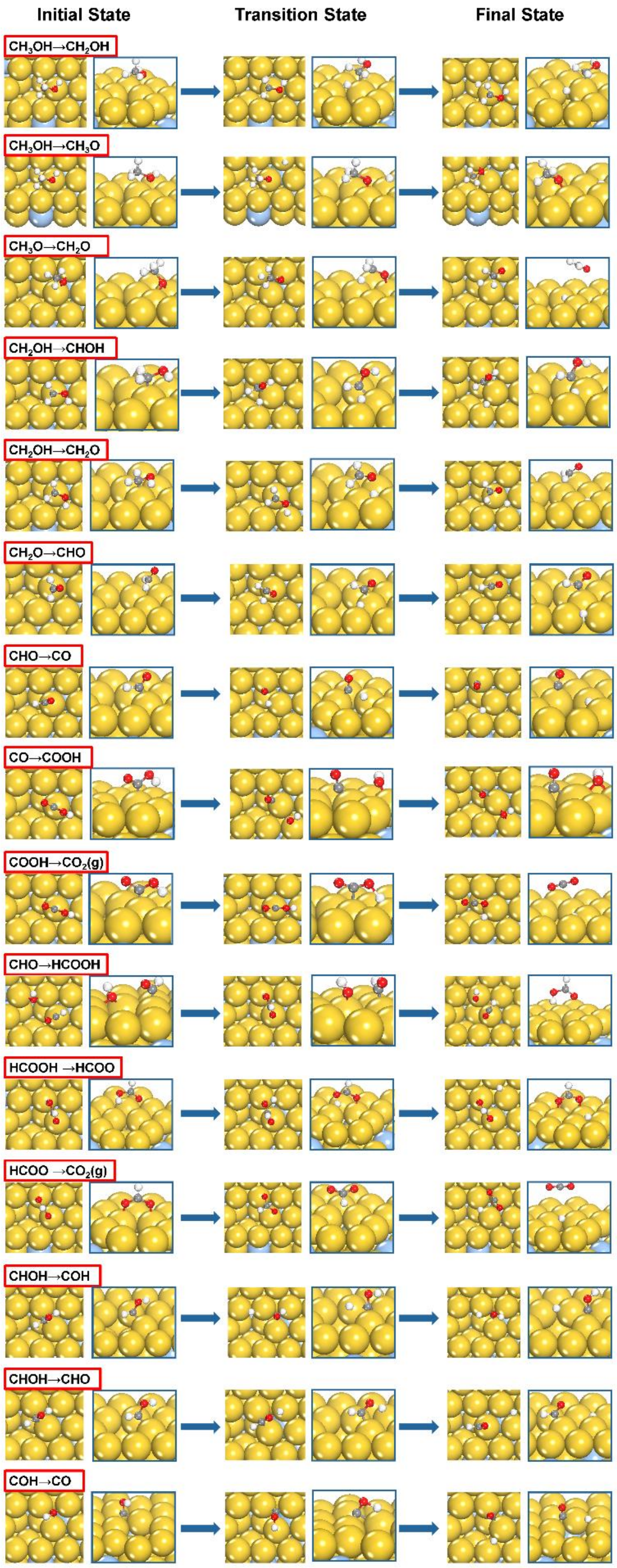

Supplementary Figure 23. Optimized geometries of the initial, transition, and final states of the MOR elementary steps on Au_3Ag-Au_{vac}. The Au, Ag, C, O, and H atoms are in yellow, light blue, grey, red, and white, respectively.

Supplementary Table 6 | Calculated activation barriers of the MOR elementary steps on the Pt (111) surface, including activation barriers at the equilibrium potential U_0 (E_a (U_0)) and the working potential U (0.78V) ($E_a(U)$), symmetry factor (β'), imaginary frequency (i-TS), and vibrational modes of the transition state.

Elementary step	$E_a(U_0)$ (eV)	U_0 (V)	β'	E_a (U=0.78 V) (eV)	i-TS (cm-1)	Vibrational mode
$*CH_3OH \rightarrow *CH_2OH$	0.57	0.27	0.56	0.28	852	C-H
$* CH_2OH \rightarrow *CHOH$	0.73	0.41	0.47	0.56	227	C-M
$*CHOH \rightarrow *COH$	0.55	0.20	0.45	0.29	341	C-H
$*CHOH \rightarrow *CHO$	0.14	0.20	0.55	-0.18	789	O-H
$*CHO \rightarrow *HCOOH$	0.42	1.23	0.49	0.64	273	C-O
$*HCOOH \rightarrow *HCOO$	0.51	0.17	0.59	0.15	253	O-H
$*HCOO \rightarrow CO_2(g)+*$	0.98	-0.00	0.67	0.46	193	O-M
$*CH_2OH \rightarrow * CH_2O$	0.74	0.17	0.53	0.42	598	O-H
$*COH \rightarrow *CO$	0.79	0.23	0.60	0.46	1527	O-H
$*CH_3OH(l) \rightarrow *CH_3O$	0.73	0.17	0.59	0.37	263	O-H
$*CH_3O \rightarrow *CH_2O$	0.11	0.58	0.44	0.02	604	C-H
$*CH_2O \rightarrow *CHO$	0.10	0.25	0.48	-0.15	343	C-H
$*CHO \rightarrow *CO$	0.19	0.17	0.52	-0.12	471	C-H
$*CO \rightarrow *COOH$	0.36	1.23	0.49	0.58	231	C-O
$*COOH \rightarrow CO_2(g)+*$	0.56	0.06	0.51	0.19	343	O-H

Supplementary Table 7| Calculated activation barriers of the MOR elementary steps on the Au_3Ag-Au_{vac} surface, including activation barriers at the equilibrium potential U_0 (E_a (U_0)) and the working potential U (0.92V) (E_a(U)), symmetry factor (β'), imaginary frequency (i-TS), and vibrational modes of the transition state.

Elementary step	$E_a(U_0)$ (eV)	U_0 (V)	β'	E_a (U=0.92 V) (eV)	i-TS (cm-1)	Vibrational mode
$*CH_3OH \rightarrow *CH_2OH$	1.25	-0.38	0.56	0.52	770	C-H
$* CH_2OH \rightarrow *CHOH$	0.92	-0.36	0.47	0.32	910	C-H
$*CHOH \rightarrow *COH$	1.56	-0.34	0.45	0.99	779	C-H
$*CHOH \rightarrow *CHO$	0.68	-0.34	0.55	-0.02	388	O-H
$*CHO \rightarrow *HCOOH$	0.19	1.06	0.49	0.26	178	C-O
$*HCOOH \rightarrow *HCOO$	1.10	-0.51	0.59	0.26	98	O-H
$*HCOO \rightarrow CO_2(g)+*$	1.23	-0.17	0.67	0.50	326	O-M
$*CH_2OH \rightarrow * CH_2O$	0.83	-0.40	0.53	0.13	207	O-H
$*COH \rightarrow *CO$	1.06	-0.39	0.60	0.26	1629	O-H
$*CH_3OH(l) \rightarrow *CH_3O$	1.75	-0.49	0.59	0.90	126	O-H
$*CH_3O \rightarrow *CH_2O$	0.38	-0.19	0.44	-0.10	563	C-H
$*CH_2O \rightarrow *CHO$	0.65	-0.37	0.48	0.03	405	C-H
$*CHO \rightarrow *CO$	0.42	-0.39	0.52	-0.26	736	C-H
$*CO \rightarrow *COOH$	0.17	1.06	0.49	0.24	129	C-O
$*COOH \rightarrow CO_2(g)+*$	0.68	-0.22	0.51	0.10	710	H-M

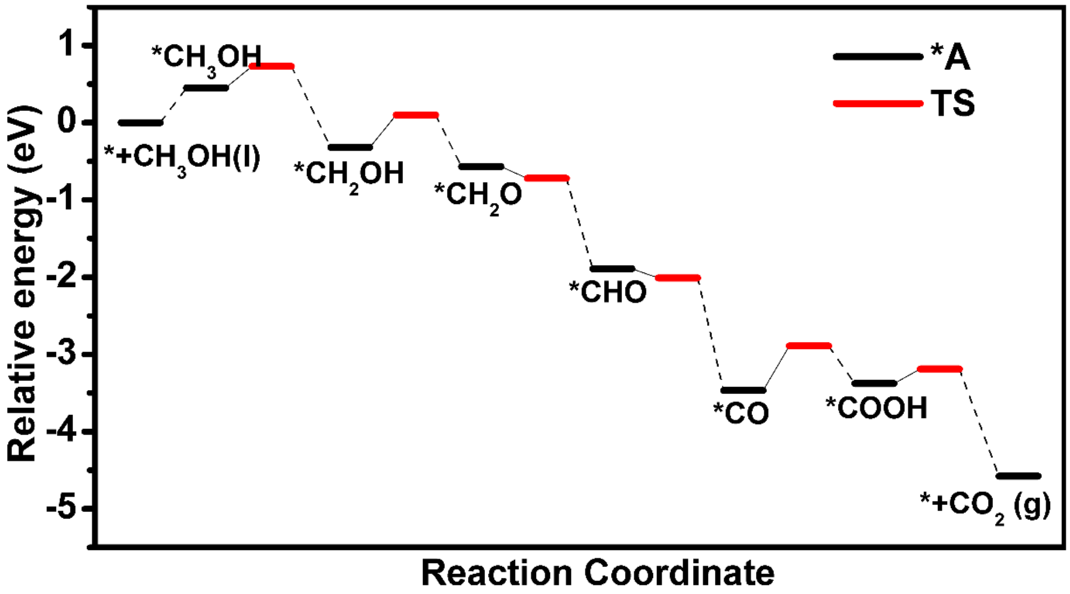

Supplementary Figure 24. Relative free energy diagram of the optimal MOR pathway on Pt (111) at the working potential of 0.78 V.

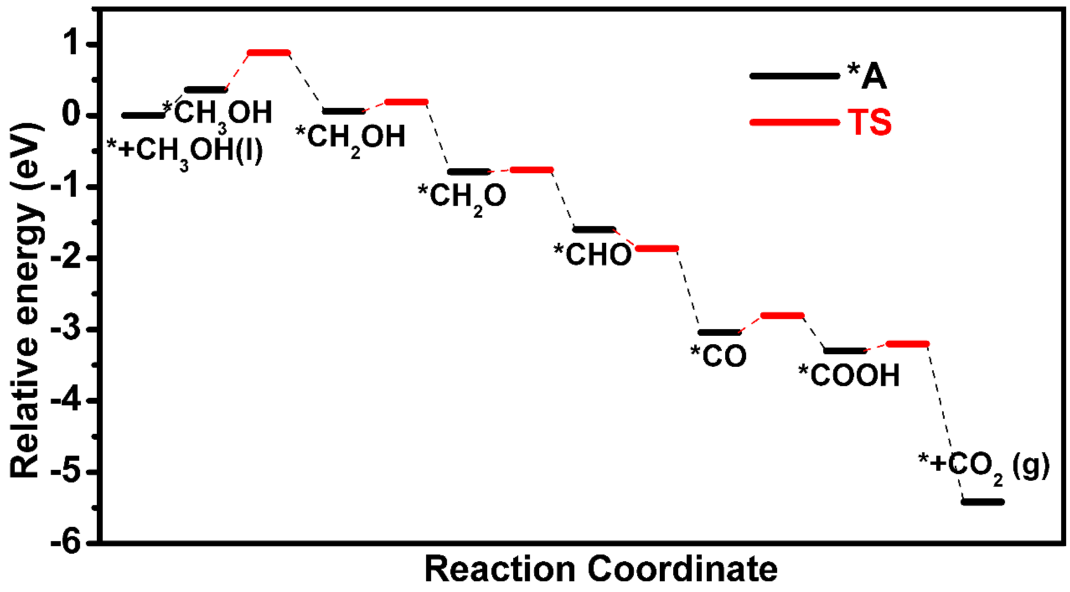

Supplementary Figure 25. Relative free energy diagram of the optimal MOR pathway on Au_3Ag-Au_{vac} at the working potential of 0.92 V.

Supplementary Table 8 | Comparison of the MOR performance of Au_3Ag NFs with other Au-based catalysts in literature

**Materials**	Electrolyte	Methanol oxidation peak potential	Mass activities	Specific activities	Electroactive Surface area	**Ref.**
**Au_3Ag NFs**	0.5M KOH + 2M CH_3OH	**- 0.06 V** (vs. Ag/AgCl)	**950** mA/mg	**3.42 mA/cm^2**	**27.8 m^2/g**	**This work**
**AuAg networks**	0.5M KOH + 2M CH_3OH	0.2 V (vs. Ag/AgCl)	19.2 mA/mg	0.33 mA/cm^2	5.82 m^2/g	10
**Nanoporous Au NPs**	1M KOH + 1M CH_3OH	0.26 V (vs. Hg/HgO)	/	0.075 mA/cm^2	/	11
**Nanoporous Au NPs**	0.5M KOH + 2M CH_3OH	0.19 V (vs. Ag/AgCl)	16.8 mA/mg	0.189 mA/cm^2	8.91 m^2/g	12
**Nanoporous Au NWs**	0.5M KOH + 1M CH_3OH	0.24 V (vs. Hg/HgO)	/	0.06 mA/cm^2 (current density)	/	13
**Au Clusters/C**	0.5M KOH + 2M CH_3OH	0.21 V (vs. Ag/AgCl)	11.2 mA/mg	0.072 mA/cm^2	15.54 m^2/g	14
**Dendritic Au**	0.1M KOH + 1M CH_3OH	0.377 V (vs. Ag/AgCl)	/	0.095 mA/cm^2 (current density)	/	15
**Au NPs/LDH**	0.25M KOH + 0.5M CH_3OH	0.08 V (vs. Ag/AgCl)	/	0.1 mA/cm^2 (current density)	/	16
**Au NPs/rGO**	0.1M KOH + 5M CH_3OH	0.333 V (vs. Hg/HgO)	34 mA/mg	/	/	17
**Au NPs/C**	0.1M KOH + 5M CH_3OH	0.355 V (vs. Hg/HgO)	48.6 mA/mg	/	/	18
**Au NPs/graphene**	0.1M KOH + 1 M CH_3OH	0.35 V (vs. Hg/HgO)	90.0 mA/mg	/	/	1
**Au nanowires**	1M NaOH + 2 M CH_3OH	0.3 V (vs. Hg/HgO)	0.9 mA/mg	/	/	19
**Au nanotubes**	0.5M KOH + 2 M CH_3OH	0.25 V (vs. Ag/AgCl)	23 mA/mg	0.14 mA/cm^2	16.25 m^2/g	20
**Au nanoballs**	0.5M KOH + 2 M CH_3OH	0.24 V (vs. Ag/AgCl)	24 mA/mg	0.23 mA/cm^2	10.34 m^2/g	21

Supplementary References

1 Choi, Y., Gu, M., Park, J., Song, H.-K. & Kim, B.-S. Graphene Multilayer Supported Gold Nanoparticles for Efficient Electrocatalysts Toward Methanol Oxidation. *Adv. Energy. Mater.* **2**, 1510-1518 (2012).

2 Kresse, G. & Furthmuller, J. Efficient iterative schemes for ab initio total-energy calculations using a plane-wave basis set. *Phys. Rev. B.* **54**, 11169-11186 (1996).

3 Blöchl, P. E. Projector augmented-wave method. *Phys. Rev. B.* **50**, 17953-17979 (1994).

4 Perdew, J. P., Burke, K. & Ernzerhof, M. Generalized gradient approximation made simple. *Phys. Rev. Lett.* **77**, 3865-3868 (1996).

5 Monkhorst, H. J. & Pack, J. D. Special points for Brillouin-zone integrations. *Phys. Rev. B.* **13**, 5188-5192 (1976).

6 Henkelman, G., Uberuaga, B. P. & Jónsson, H. A climbing image nudged elastic band method for finding saddle points and minimum energy paths. *J. chem. phys*. **113**, 9901-9904 (2000).

7 Heyden, A. Bell, A. T. & Keil, F. J. Efficient methods for finding transition states in chemical reactions: Comparison of improved dimer method and partitioned rational function optimization method. *J. chem. phys*. **123**, 224101 (2005).

8 Nørskov, J. K. *et al.* Origin of the overpotential for oxygen reduction at a fuel-cell cathode. *J. Phys. Chem. B.* **108**, 17886-17892 (2004).

9 Nie, X. *et al*. Selectivity of CO2 reduction on copper electrodes: the role of the kinetics of elementary steps. *Angew. Chem. Int. Edit.* **52**, 2459-2462 (2013).

10 Fang, J. *et al.* A general soft-enveloping strategy in the templating synthesis of mesoporous metal nanostructures. *Nat. Commun.* **9**, 521 (2018).

11 Graf, M., Haensch, M., Carstens, J., Wittstock, G. & Weissmuller, J. Electrocatalytic methanol oxidation with nanoporous gold: microstructure and selectivity. *Nanoscale.* **9**, 17839-17848 (2017).

12 Pedireddy, S. *et al.* One-step synthesis of zero-dimensional hollow nanoporous gold nanoparticles with enhanced methanol electrooxidation performance. *Nat.*

Commun. **5**, 4947 (2014).

13 Li, H., Li, Y.-J., Sun, L.-L. & Zhao, X.-L. One-step, template-free electrochemical preparation of three-dimensional porous Au nanowire network and its enhanced activity toward methanol electrooxidation. *Electrochim. Acta.* **108**, 74-78 (2013).

14 Mao, Z. *et al.* Confining Gold Nanoclusters in Highly Defective Graphitic Layers To Enhance the Methanol Electrooxidation Reaction. *ChemCatChem.* **10**, 141-147 (2018).

15 Han, X., Wang, D., Huang, J., Liu, D. & You, T. Ultrafast growth of dendritic gold nanostructures and their applications in methanol electro-oxidation and surface-enhanced Raman scattering. *J. Colloid. Interf. Sci.* **354**, 577-584 (2011).

16 Wang, Y., Zhang, D., Tang, M., Xu, S. & Li, M. Electrocatalysis of gold nanoparticles/layered double hydroxides nanocomposites toward methanol electro-oxidation in alkaline medium. *Electrochim. Acta.* **55**, 4045-4049 (2010).

17 Guo, Y. *et al.* One pot preparation of reduced graphene oxide (RGO) or Au (Ag) nanoparticle-RGO hybrids using chitosan as a reducing and stabilizing agent and their use in methanol electrooxidation. *Carbon.* **50**, 2513-2523 (2012).

18 Yan, S., Zhang, S., Lin, Y. & Liu, G. Electrocatalytic Performance of Gold Nanoparticles Supported on Activated Carbon for Methanol Oxidation in Alkaline Solution. *J. Phys. Chem. C.* **115**, 6986-6993 (2011).

19 Guo, T. *et al.* Synthesis of Ultralong, Monodispersed, and Surfactant-Free Gold Nanowire Catalysts: Growth Mechanism and Electrocatalytic Properties for Methanol Oxidation Reaction. *J. Phys. Chem. C.* **121**, 3108-3116 (2017).

20 Yin, X., Teradal, N. L. & Jelinek, R. Porous Gold Nanotubes for Enhanced Methanol Oxidation Catalysis. *ChemistrySelect.* **2**, 10961-10964 (2017).

21 Yin, X., Teradal, N. L., Morag, A. & Jelinek, R. Catalytic Au Wool-Ball-Shaped Nanostructures. *ChemCatChem.* **9**, 2473-2479 (2017).
